# Computing disease-linked SOD1 mutations: deciphering protein stability and patient-phenotype relations

**DOI:** 10.1038/s41598-017-04950-9

**Published:** 2017-07-05

**Authors:** Vijay Kumar, Safikur Rahman, Hani Choudhry, Mazin A. Zamzami, Mohammad Sarwar Jamal, Asimul Islam, Faizan Ahmad, Md. Imtaiyaz Hassan

**Affiliations:** 1 Centre for Interdisciplinary Research in Basic Sciences, Jamia Millia Islamia, New Delhi, 110025 India; 2 Department of Medical Biotechnology, Yeungnam University, Gyeongsan, 712-749 South Korea; 3 Department of Biochemistry, Cancer Metabolism and Epigenetic Unit, Faculty of Science, Center of Innovation in Personalized Medicine, Cancer and Mutagenesis Unit, King Fahd Center for Medical Research, King Abdulaziz University, Jeddah, Saudi Arabia; 4 Department of Biochemistry, Cancer Metabolism and Epigenetic Unit, Faculty of Science, Cancer and Mutagenesis Unit, King Fahd Center for Medical Research, King Abdulaziz University, Jeddah, Saudi Arabia; 5 King Fahd Medical Research Center, King Abdulaziz University, P.O. Box 80216, Jeddah, 21589 Saudi Arabia

## Abstract

Protein stability is a requisite in the field of biotechnology, cell biology and drug design. To understand effects of amino acid substitutions, computational models are preferred to save time and expenses. As a systemically important, highly abundant, stable protein, the knowledge of Cu/Zn Superoxide dismutase1 (SOD1) is important, making it a suitable test case for genotype-phenotype correlation in understanding ALS. Here, we report performance of eight protein stability calculators (PoPMuSiC 3.1, I-Mutant 2.0, I-Mutant 3.0, CUPSAT, FoldX, mCSM, BeatMusic and ENCoM) against 54 experimental stability changes due to mutations of SOD1. Four different high-resolution structures were used to test structure sensitivity that may affect protein calculations. Bland-Altman plot was also used to assess agreement between stability analyses. Overall, PoPMuSiC and FoldX emerge as the best methods in this benchmark. The relative performance of all the eight methods was very much structure independent, and also displayed less structural sensitivity. We also analyzed patient’s data in relation to experimental and computed protein stabilities for mutations of human SOD1. Correlation between disease phenotypes and stability changes suggest that the changes in SOD1 stability correlate with ALS patient survival times. Thus, the results clearly demonstrate the importance of protein stability in SOD1 pathogenicity.

## Introduction

Protein stability is a fundamental property affecting proteins function, activity, and regulation. It plays a major role in evolution, industrial applications and many diseases^1–7^. For the last several decades there has been a growing attention to understand biophysical principles behind protein stability using both the theoretical and experimental methods^8–12^. Accurate prediction of stability is important for both academic and applied research. Several methods have been developed to predict the effect of mutations on the stability of proteins. These methods can be grouped into three main categories based on the strategy used in the calculation: (a) physical energy functions, (b) potential energy functions, and (c) machine learning methods. The performances of these predictors were assessed and compared in different studies using datasets of experimentally characterized mutants^13–15^. Overall conclusions of these studies were inconsistent; all methods showed a correct trend in the predictions but with moderate accuracies. PoPMuSiC was the only predictor shown to perform quite well in comparison with other methods^14^. However, predictions of the stability of protein variants are challenged by noise in structural data and experimental measurements as well as the complex physics of protein folding and stability. To highlight the importance of protein structures for assessing protein stability, recent work has addressed benchmarks of protein stability data where structures were in the near-atomic-resolution range, to minimize structural noise^16, 17^.

Cu/Zn superoxide dismutase (SOD1) is an extensively studied metalloenzyme that has become a paradigm for understanding protein folding and misfolding associated with motor neuron disease, amyotrophic lateral sclerosis (ALS)^18^. ALS is a fatal neurodegenerative disease that causes progressive paralysis and death within 3–5 years of diagnosis^19, 20^. SOD1 is a homodimer with 153 amino acid residues in each monomer that catalyzes the metal dependent dismutation of superoxide anions (O_2_^−^) to hydrogen peroxide (H_2_O_2_) and oxygen (O_2_). Each chain forms an eight-stranded β-barrel that contains one intramolecular disulfide bond and binds zinc and copper ions. The eight strands are connected by two long and functionally important loops. An electrostatic loop VII (residues 121−142), provides guidance of the superoxide substrate toward the catalytic copper site^21^, while the other is the zinc-binding loop IV (residues 49−83). The zinc site is important for the structure and function of the active copper site^22^.

Mutations in SOD1 account for ~20% of all familial ALS (fALS) cases or about 2% of all ALS occurrences^19^. More than 150 mutations have been identified in ALS patients^23^. These mutations result in structural destabilization, metal depletion, reduction of disulfide bonds and alterations in the functional properties of SOD1, and thus play a crucial role in the formation of toxic aggregates and in the pathology of ALS^24–33^. Despite its pathological importance, there has been very little theoretical study to understand the stability effects of SOD. Recently, Kepp made a significant attempt to evaluate the relative performance of theoretical methods for calculating the stability of SOD1^16^ and myoglobin^17^ variants. The relative performance of these methods was not very structure-dependent.

The present work extends the previous benchmark study for SOD1 stability^16^ using three different structures used previously. Moreover, in this work we have also tried to establish a relationship between protein stability and patient phenotype seen in ALS. Findings of this study may prove to be useful as a diagnostic and/or predictive tool to assess effects of mutations by relating the critical stages of disease development to alterations in SOD1 stability.

## Results and Discussion

### Correlations between experimental holodimer and apo-monomer data

Experimentally determined ΔΔ*G* values for both the apo-monomer and holodimer SOD1 (Table S1) are compared as described previously^16^. Figure S1 shows that the correlation coefficient (R^2^) is 0.70. A correlation coefficient of 1.0 would imply that the effects of the mutations are mainly additive and the monomers would be non-interacting. This result (R^2^ = 0.70) suggests that using monomer stability effects, we can assess the stability of the biologically relevant functional dimer.

### Performance of the methods for monomer SOD1 data: Correlation to experimental data

**Figure 1:**
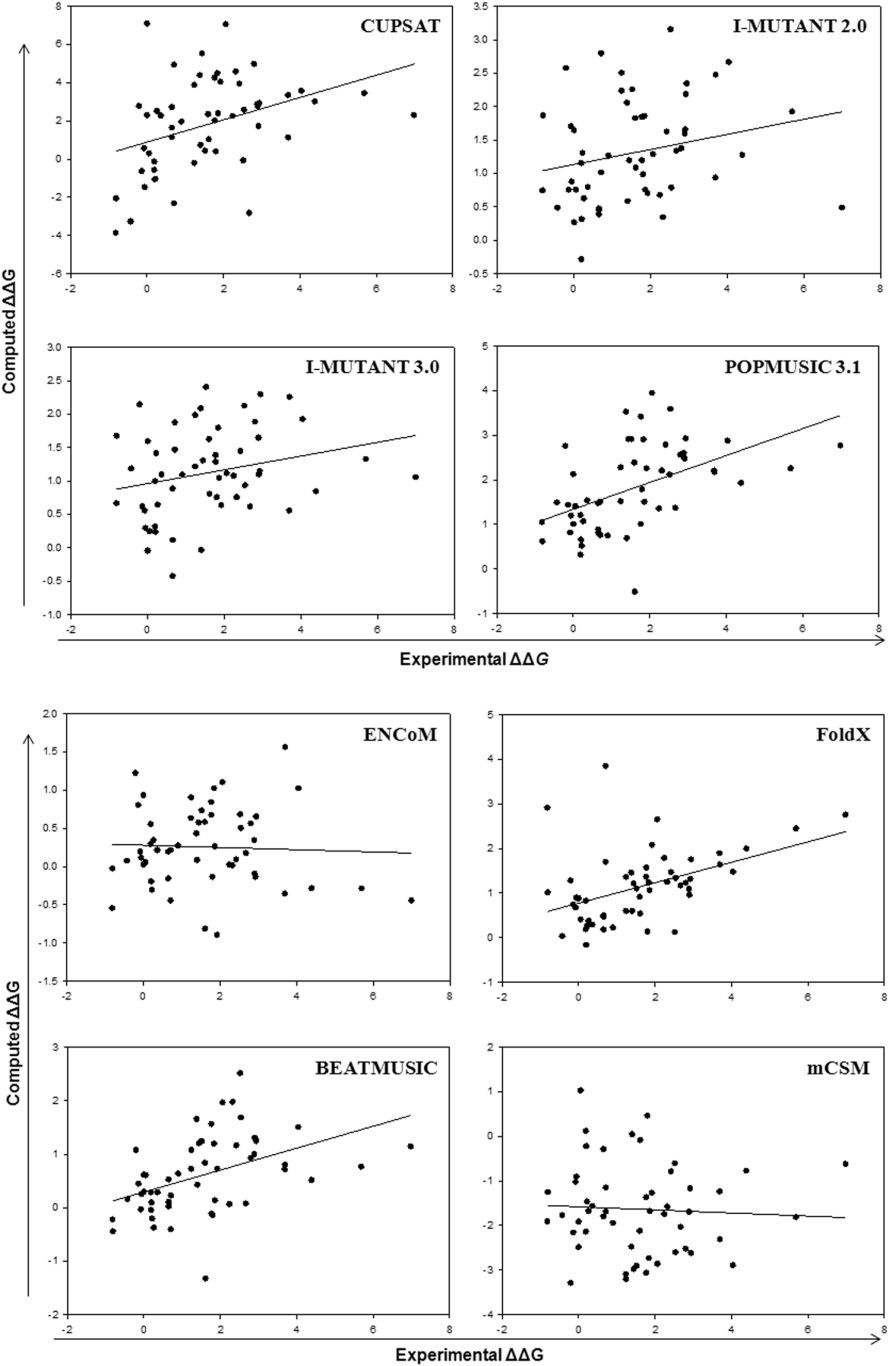
Correlation between experimental monomer and computed stability changes, ΔΔG (kcal/mol) for 54 mutants using 2C9V and eight methods.

**Figure 2:**
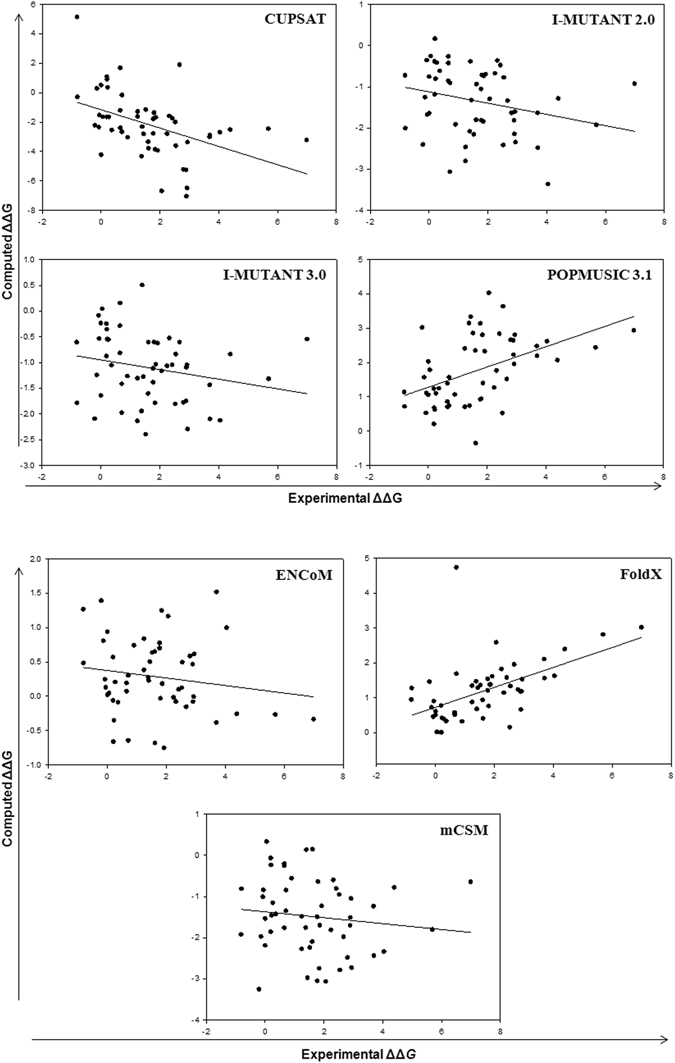
Correlation between experimental monomer and computed stability changes, ΔΔG (kcal/mol) for 54 mutants using 2XJK and eight methods. (Data for other structures has been shown in Supporting Information).

Numerical values for the stability changes from eight computational predictors in the four different crystal structures are compiled in supplementary information (Tables S2–S5). In Table 1, three performance indicators as discussed in Methods, are shown for the eight methods applied to four crystal structures. The plots resulting from linear regression for 2C9V representing the physiological holodimeric structure of SOD1 of high resolution (1.07 Å) with only one dimer per structure file, are shown in Fig. 1, and those for 2XJK (1.45 Å) representing the real functional monomer are shown in Fig. 2. Such plots for loop-less SOD1, 4BCZ (1.93 Å) and apo dimer, 1HL4 (1.82 Å) are provided in supporting Figs S1 and S2, respectively.

**Table 1:** Overall performance of stability predictors for 54 SOD1 monomer mutants using four different crystal structures.

Methods	Metrics^a^	2C9V	1HL4	2XJK	4BCZ	Average
CUPSAT	MAE	1.81	2.3	4.18	4.05	3.09
	MSE	−0.23	−0.52	3.8	3.61	1.66
	R	0.38	0.27	0.45	0.3	0.35
	R^2^	0.14	0.07	0.2	0.09	0.12
I-Mutant 2.0	MAE	1.17	1.15	2.97	2.62	1.97
	MSE	0.28	0.29	2.96	3.24	1.69
	R	0.23	0.23	0.26	0.23	0.23
	R^2^	0.05	0.05	0.07	0.05	0.05
I-Mutant 3.0	MAE	1.17	1.16	3.72	2.3	2.08
	MSE	0.46	0.48	2.72	2.93	1.64
	R	0.25	0.23	0.22	0.16	0.21
	R^2^	0.06	0.05	0.05	0.03	0.05
PoPMuSiC 3.1	MAE	1.08	1.08	1.12	0.98	1.06
	MSE	−0.23	−0.22	−0.13	0.17	−0.1
	R	0.5	0.5	0.47	0.55	0.5
	R^2^	0.25	0.25	0.22	0.3	0.25
ENCoM	MAE	1.54	1.6	1.65	1.7	1.62
	MSE	1.33	1.39	1.34	1.56	1.4
	R	0.04	0.01	0.16	0.04	0.06
	R^2^	0.002	0	0.02	0.002	0.01
FoldX	MAE	1.1	1.1	1.04	1.1	1.08
	MSE	0.45	0.56	0.45	0.78	0.55
	R	0.45	0.45	0.53	0.53	0.49
	R^2^	0.2	0.2	0.28	0.28	0.24
BeatMusic	MAE	1.17	1.19		1.41	1.26
	MSE	0.97	0.94		1.31	1.07
	R	0.45	0.42		0.46	0.44
	R^2^	0.2	0.17		0.21	0.19
mCSM	MAE	3.26	3.14	3.12	3.24	3.19
	MSE	3.22	3.12	3.11	3.22	3.16
	R	0.05	0.11	0.12	0.24	0.13
	R^2^	0.03	0.01	0.01	0.06	0.03

^a^MAE: Mean absolute error (kcal/mol). MSE: Mean signed error (experimental value minus computed value) in kcal/mol. R & R^2^ = Correlation coefficient from regression analysis.

Results for the representative and highest-resolution of holodimer SOD1 structure, 2C9V are shown in Fig. 1. This structure has all 153 residues present and gives the largest correlation coefficients. Notably, PoPMuSiC 3.1 displayed an R^2^ value of 0.25, corresponding to R value of 0.5, and considerably better than any of the other six methods. It was followed by Fold X (R = 0.45) and BeatMusic (R = 0.45), ENCoM (R = 0.4), CUPSAT (R = 0.37), I-Mutant 3.0 (R = 0.24), I-Mutant 2.0 (R = 0.23) and mCSM (R = 0.05). For the representative apo-monomer SOD1structure, 2XJK (Fig. 2), Fold X displayed an R value of 0.53 followed by PoPMuSiC 3.1 (R = 0.47), CUPSAT (R = 0.45), I-Mutant 2.0 (R = 0.26), I-Mutant 3.0 (R = 0.22), ENCoM (R = 0.16) and mCSM (R = 0.12) (Table 1). Results for 4BCZ (1.93 Å) are also shown in Fig. S2. This structure has 110 residues present and loops IV and VII were substituted with short Gly-Ala-Gly linkers^34^. It has been shown that removal of the loops in SOD1 leads to soluble, monomeric β-barrels with increase in stability^34^. Interestingly, in several cases, the overall agreement with experimental data was reasonably similar to realistic dimeric SOD1 (2C9V). The descending trend in R values is: PoPMuSiC 3.1 (0.55) ~ Fold X (0.53) > BeatMusic (0.46) > CUPSAT (0.3) > mCSM (0.24) ~ I-Mutant 2.0 (0.23) > I-Mutant 3.0 (0.16) ≫ ENCoM (0.04) (Table 1).

In the independent benchmark against 2156 experimentally derived data points by Potapov *et al*.^13^, six methods displayed correlation coefficients R from 0.26 (Rosetta) to 0.59 (EGAD). Among the methods studied in the present work, only FoldX and I-Mutant 2.0 were also studied by Popatov *et al*.^13^ and had R ~ 0.5 in both cases. The correlation coefficient of 0.53 with FoldX found for apomonomer and loop less SOD1 mutations are thus similar to what was expected. The correlation coefficient (0.23–0.26) in case of I-Mutant 2.0 is substantially lower than Popatov *et al*.^13^ but similar to what was expected in dimeric SOD1 structure^16^.

These results suggest that PoPMuSiC 3.1 and Fold X produce a reasonable correlation. Thus, PoPMuSiC, which is based on environment-dependent substitution frequencies, and which correlate with the chemical properties of the amino acids, was most accurate in this study.

### Bland Altman Analysis

A Bland-Altman (BA) plot was used to analyze the agreement between the experimental and computed stability values. The mean difference (i.e. bias) between the stability values indicates the overall bias present in the data, while the limits of agreement (Mean ± 1.96 SD) indicate the precision of the computations. The BA test is a statistically robust method of accessing reliability and agreement between two quantitative measurements.

Results for the overall bias between experimental and computed stability values are reported in Table S6. A bias close to zero suggests that there are negligible differences between the experiment and predicted stability values. Whereas, negative bias implies that the stability predictor method estimates less i.e. underreport as compared to the experimental value. Globally, PoPMuSiC showed better features of reliability irrespective of the structure (bias value in the range of −0.17 to 0.24). On the other hand, mCSM (bias: −3.1 to −3.5) and ENCoM (bias: −1.3 to −1.5) showed lower values of bias for all the structures. Moreover, except CUPSAT and I-Mutant 2.0/3.0, all other stability predictors are structure insensitive and correlate well for the structures. However, the variability between the two methods is well within the range of estimated 95% confidence limit (Table S6). Thus, stability predicted by PoPMuSiC exhibits strong correlations, along with low bias values, when compared with the experimental value.

### Mean absolute errors

Table 1 lists MAE, MSE, R and R^2^ values for all eight methods applied to the four structures. For holodimer SOD1, the methods ordered according to their ascending MAEs is: PoPMuSiC 3.1 (1.08 kcal/mol) < FoldX (1.1 kcal/mol) < I-Mutant 2.0 (1.17 kcal/mol) ~ I-Mutant 3.0 (1.17 kcal/mol) ~ BeatMusic (1.17 kcal/mol) < ENCoM (1.5 kcal/mol) < CUPSAT (1.81 kcal/mol) ≪ mCSM (3.2 kcal/mol). This order resembles what has been reported earlier by Kepp^16, 17^, though he didn’t include all these predictors.

In case of apo-monomer SOD1 having single copy (2XJK), the methods order according to ascending MAEs is: FoldX (1.04 kcal/mol) < PoPMuSiC 3.1 (1.11 kcal/mol) < ENCoM (1.6 kcal/mol) ≪ I-Mutant 2.0 (2.9 kcal/mol) < mCSM (3.1 kcal/mol) ≪ I-Mutant 3.0 (3.7 kcal/mol) ≪ CUPSAT (4.18 kcal/mol). For Loopless SOD1 having two chains (4BCZ), the order is similar to that seen for apo-monomer mutations except mCSM and I-Mutant 3.0 which have shifted positions (see Table 1). The performance of mCSM is quite good as compared to CUPSAT in the case of apo-monomer and loop-less SOD1, while the opposite is true in the case of holodimer SOD1. Good performance of PoPMuSiC 3.1 and I-Mutant 3.0 over CUPSAT was also seen previously^16, 17^. Moreover, PoPMuSiC 3.1 performs better than both I-Mutant 2.0 and I-Mutant 3.0, which is not the case in myoglobin as studied^17^.

These results thus suggest that a method can provide a very good trend in predicted stabilities without actually being numerically accurate, as R and MAE are independent characteristics of the statistics of the methods. Overall, the accuracy of the selected methods in this work is encouraging.

### Systematic errors of the methods

Table 1 also lists the MSEs for each method applied to each of the four structures. These values are a measure of the overall bias of each method towards stabilization or destabilization. A positive value means that the method predicts, on the average, more stabilization as compared to the experimental data. Following can also be seen in this table. (a) For holodimer SOD1 having single copy of dimer (2C9V) the order is: CUPSAT (−0.23 kcal/mol) ~ PoPMuSiC 3.1 (−0.23 kcal/mol) ≪ I-Mutant 2.0 (0.28 kcal/mol) < FoldX (0.45 kcal/mol) ~ I-Mutant 3.0 (0.46 kcal/mol) ≪ BeatMusic (0.97 kcal/mol) ≪ ENCoM (1.33 kcal/mol) ≪mCSM (3.22 kcal/mol). (b) In case of 1HL4, the trend is similar to that of 2C9V except that CUPSAT and PoPMuSiC 3.1 have exchanged their places. (c) In case of 2XJK, the MSEs increased as PoPMuSiC 3.1 (−0.13 kcal/mol) ≪ FoldX (0.45 kcal/mol) ≪ ENCoM (1.34 kcal/mol) ≪ I-Mutant 3.0 (2.72 kcal/mol) < I-Mutant 2.0 (2.96 kcal/mol) ≪ mCSM (3.11 kcal/mol) ≪ CUPSAT (3.8 kcal/mol). (d) For 4BCZ, the order is: PoPMuSiC 3.1 (0.17 kcal/mol) < FoldX (0.78 kcal/mol) < BeatMusic (1.31 kcal/mol) < ENCoM (1.56 kcal/mol) ≪ I-Mutant 3.0 (2.93 kcal/mol) < mCSM (3.22 kcal/mol) ~ I-Mutant 3.0 (3.24 kcal/mol) < CUPSAT (3.61 kcal/mol).

Thus, mCSM displayed a large systematic error (MSE > 3.0 kcal/mol) suggesting that it is most stabilizing for SOD1 mutations. However, the systematic bias toward destabilization/stabilization compared to the experimental data is relatively small for PoPMuSiC 3.1 and FoldX for all four structures studied. The overall numerical accuracy measured by MAE, is: PoPMuSiC > FoldX > BeatMusic > ENCoM > I-Mutant2.0 > I-Mutant3.0 ≫ CUPSAT > mCSM. In terms of systematic error, the order of increasing MSE is: PoPMuSiC < FoldX < BeatMusic < ENCoM < I-Mutant3.0 < CUPSAT < I-Mutant2.0 < mCSM. For stability trend, measured by R value, the performance is: PoPMuSiC~ FoldX > BeatMusic > CUPSAT > I-Mutant2.0 ~ I-Mutant3.0 > mCSM > ENCoM. In this ranking, the position of CUPSAT was very dependent on the structure used.

This overall performance of metrics suggests that the metrics are not well correlated in this work. CUPSAT show good stability trend but with large numerical error, while I-Mutant 2.0/I-mutant 3.0 has poor trend and large systematic errors but shows high numerical accuracy. A better stability trend with high numerical accuracy and small systematic errors can be obtained as seen in the case of PoPMuSiC 3.1 and FoldX, thus providing realistic, narrow distributions of mutation effects.

Thus, the comparison provides insight into the strengths and weaknesses of such methods, as described by the three metrics studied. The qualitative physics of the models, as explained by the correlations, are similar and of modest accuracy, whereas they are differently biased in terms of systematic errors, and CUPSAT along with I-mutant 2.0/3.0 lacks the numerical scaling of a realistic distribution of such stability effects, having wide numerical range.

### Performance of the methods for holodimer SOD1 Data: Correlation to experimental data

We have also compared the performance of the eight methods against experimental dimer data set of 33 single-point mutations and the result are shown in Table S7. These results reveal that the trends are similar to or even more accurate in some cases when compared to the monomer data suggesting that the physics of the different mutations remain unaffected by the dimer/monomer structure. The plots resulting from linear regression for four structures are shown in Fig. S4.

Previously, Kepp showed that the additivity of effects of mutation works well when describing performance of the computed methods^16, 17^. We have also shown that this is in good agreement with other predictors. Table S7 shows the main metrics of each method applied to each of the four structures. As seen in case of the most realistic dimer structure 2C9V, PoPMuSiC 3.1 performs quite well (R = 0.51, MAE = 0.65 kcal/mol and MSE = 0.76 kcal/mol). Moreover, CUPSAT performs quite well in its trend with R = 0.38, despite large numerical errors due to its stability bias and broad range of ΔΔG values. ENCoM (R = 0.001), mCSM (R = 0.11) and FoldX (R = 0.18) provide a poor trend on the dimer data set.

In case of apomonomer structure, CUPSAT correlates well with dimer data (R = 0.53) despite most destabilizing (MSE = 3.73 kcal/mol) and large spread in its ΔΔG values (MAE = 3.21 kcal/mol). For loopless SOD1 structure, the trend is similar to that of dimer SOD1 with PoPMuSiC 3.1 performs well (R = 0.45, MAE = 0.61 kcal/mol and MSE = 1.21 kcal/mol).

These results suggest that the mutations are largely independent of the dimerization state of the protein.

### Analysis of complicated sites

**Figure 3:**
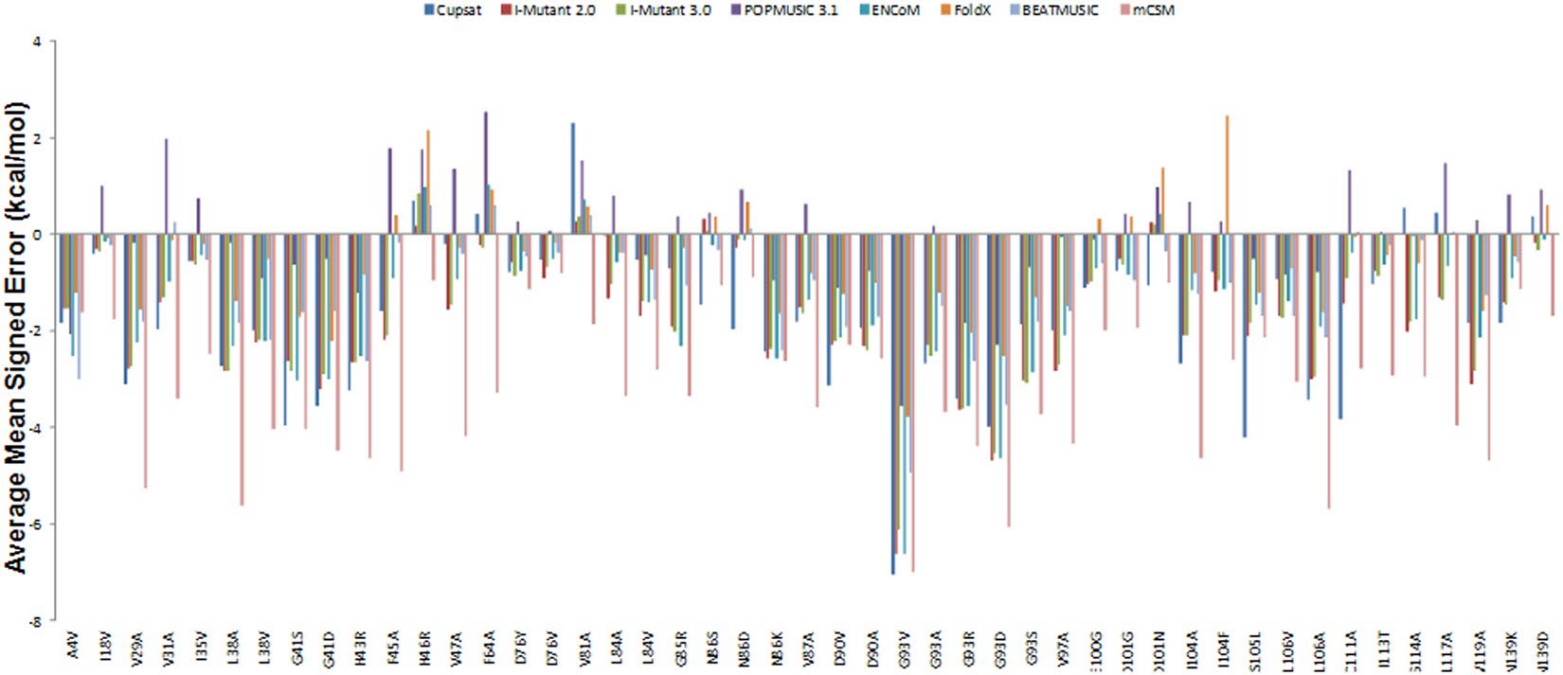
Analysis of complicated sites. Mean signed errors (kcal/mol) averaged over all four structures for each eight methods, decomposed into individual mutations.

Next, we sought to know how the performance of the methods depends on the specific sites in the protein. This will allow us to identify sites that are poorly described by methods used. Figure 3 shows MSEs of all eight methods averaged over all four structures, compared to the monomer experimental data and disintegrated into individual 47 mutations. As can be seen in this figure, methods tend to perform poorly at all of the sites and except, H46R, F64A and V81A, mutations at many sites show too much stabilization.

**Figure 4:**
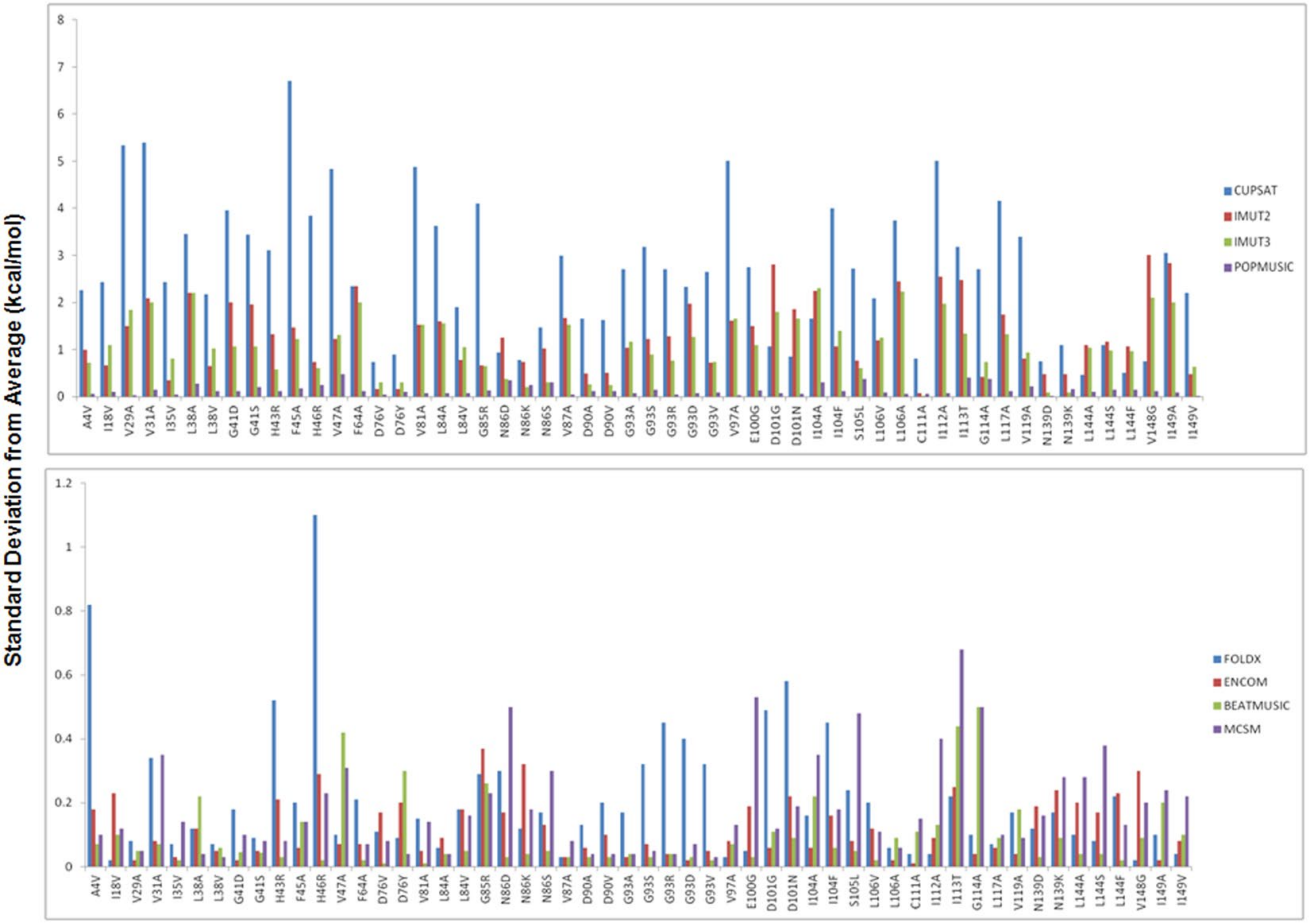
Structural sensitivity of methods decomposed into individual mutants (standard deviation from average result (kcal/mol) using the four structures. Top: Methods previously studied by Kepp^16^. Bottom: FoldX, ENCoM, BeatMusic, and mCSM used in this study. The total data set in Supporting Information, Tables S8 and S9.

Although the average MAE is 1.92 kcal/mol; many MAE values exceed from 3.0 to 4 kcal/mol. Moreover, CUPSAT and mCSM have the largest deviations from the experiment mainly because these methods have too broad ΔΔ*G* distributions. As seen in Fig. 4, mutations involving glycine are particularly challenging for most of the methods, as also observed previously for SOD1 data set^16^. This is likely due to the low structure propensity, small size, and associated possibilities of structure changes in case of glycine. These changes cannot easily be modeled by standard methods. There are eleven glycine-involving mutations in the data set. Among the errors larger than 2.0 kcal/mol, these mutations (G41S, G41D, G85R, G93V, G93A, G93R, G93D, and G93S) are all exclusively associated with large over-stabilizations by several methods. For E100G and D101G, the error is less than 2.0 kcal/mol, but is still associated with over-stabilization.

The other difficult residue in the current data set is mutation by alanine. There are eighteen such mutations in the current data points and thus highly significant. Out of these 18 mutations, 10 mutations (V29A, V31A, L38A, F45A, D90A, G93A, V97A, I104A, L106A and V119A) are associated with over-stabilization by several methods. Furthermore, there are five mutations involving asparagine (N86S, N86D, D101N, N139K and N139D) which are noteworthy because the methods have substantially different biases toward either destabilization or stabilization in these cases. For the seven metal-adjacent sites (F45, H46, V47, F64, V81, L117 and V119), method show different biases toward stabilization (F45, V47, L117 and V119) or destabilization (H46, F64 and V81). Only, POPMUSIC 3.1 substantially over-estimates the destabilization of these sites.

Thus, PoPMuSiC, being less sensitive to structure, is the best predictor across all types of mutations, whereas FoldX and I-Mutant2.0/3.0 shows more accuracy when difficult sites are removed.

### Structural sensitivity of the methods

The sensitivity of a method’s output to the protein structure used for calculation is a major issue, for a choice of crystal structure can considerably affect the computed results^16, 17, 35, 36^. Furthermore, crystal structures vary substantially in terms of resolution and refinement quality, missing residues, presence of heteroatom, crystal symmetry and conditions (pH, salt, T), this is another major challenge to protein modelling that is not yet widely explored. Protein stability calculations seen in the literature use structures with variable R-values, missing residues, heteroatoms, special crystal symmetries or units cells, and variable conditions of salt, pH, and temperature. Benchmark studies generally rely on a large number of crystal structures of variable quality across the known protein mutation data causing noise in the data and potentially systematic and random errors. Because of these challenges, we thought that it would be worthwhile to investigate data sets where a range of high-resolution structures are available.

Recently, the sensitivity of protein stability calculators to the three-dimensional structure in case of SOD1^16^ and sperm-whale myoglobin^17^ has been studied. We extend our study in SOD1 by using four different methods in addition to the methods previously studied and using two different structures for SOD1 also. The structures of SOD1 studied here includes practical dimers with single copy (2C9V, 1.07 Å), dimmers with multiple copies (1HL4, 1.82 Å), monomer with single copy (2XJK, 1.45 Å) and loop-less SOD1 (1.93 Å). The selection of crystal structures allows an examination of the effect of different protein states, role of loops, resolution, pH, and protein-protein interactions on the quality of the structure for the specific purpose of predicting stabilities. Figure 4 shows the structural sensitivity of the methods over the four set of structures. It is evaluated by computing for each method the average standard deviation from the average ΔΔ*G* for each mutation using the four structures in the data set. The numerical values of the average ΔΔ*G* and standard deviation in ΔΔG for each mutation computed with each method are given in Tables S8 and S9, respectively. The top panel of Fig. 4 shows the result for all four methods reported previously^16^, whereas the lower panel shows the results for the other four methods used in this study. As seen in the top panel of this figure, except PoPMuSiC all three other methods, CUPSAT, I-Mutant 2.0 and I-Mutant 3.0 are very much structure sensitive. As can also be seen here, sensitivities are essentially not very residue-dependent compared to the method-dependence. The lower panel of Fig. 4 shows the results for the methods which are strikingly less sensitive than CUPSAT, I-Mutant 2.0 and I-Mutant 3.0. As seen in the figure, the standard deviations for all sites were generally smaller than 0.3 kcal/mol for ENCoM and BeatMusic. For FoldX and mCSM, only a few sites had 0.3–0.6 kcal/mol sensitivity. Sensitivity larger than 0.3 kcal/mol was found in FoldX for A4V, H43R, H46R, G93R, G93D, D101G, D101N and I104F. It was shown earlier that performance of the predictors was poor in these sites and has different biases toward either destabilization or stabilization^16^. Thus, while most computations give rise to standard deviations that are smaller than 0.5 kcal/mol, some methods, notably CUPSAT and I-Mutant, exhibit cases where the standard deviation exceeds even 2.0 kcal/mol. This means that the choice of crystal structure contributes significantly to the standard uncertainty of the protein property calculations.

When averaged over all 54 mutations, the methods have the following standard deviations, in order of increasing structure sensitivity: BeatMusic (0.09 kcal/mol) < ENCoM (0.12 kcal/mol) < PoPMuSiC 3.1 (0.14 kcal/mol) < FoldX (0.18 kcal/mol) ~ mCSM (0.18 kcal/mol) ≪ I-Mutant 3.0 (1.09 kcal/mol) < I-Mutant 2.0 (1.30 kcal/mol) ≪ CUPSAT (2.66 kcal/mol). Though, the value of standard deviation is large in this case, the order of sensitivity is consistent with the previous results^16^ except in the case of PoPMuSiC 3.1. (BeatMusic, ENCoM, FoldX and mCSM was not studied in previous work.) For the most of the cases low structure sensitivity is a clear advantage as it increases the adaptability of the method and reduces the role of noises in structure data. However, high structural sensitivity will be needed in case of high-resolution structures.

Figure S5 shows the correlation plots of ΔΔG valuesfor all possible 2907 SOD1 mutations (i.e., 19 mutationsin 153 sites) computed with PoPMuSiC 3.1, comparing with all four structure templates. The use of higher resolution dimeric structure (2C9V) and the monomeric structure (2XJK) provide thestrongest correlations (R^2^ ~ 0.85). However, if 2C9V is compared with 4BCZ, the computed output will deviate more from the results obtained with high-resolution structures (R^2^ ~ 0.3). The correlation doesn’t show any significant changes even comparing 4BCZ with 2XJK (R^2^ ~ 0.31). Also, the number of problematic outliers increases as we compared the correlation with 4BCZ as seen from the large scattering of points in Fig. S5. One should take into account that PoPMuSiC is very much structure insensitive, so the use of other methods may show much less sensitivities than those reported in Fig. S5.

### Performance of the methods for SOD1 Data: Correlation to patient data

**Figure 5:**
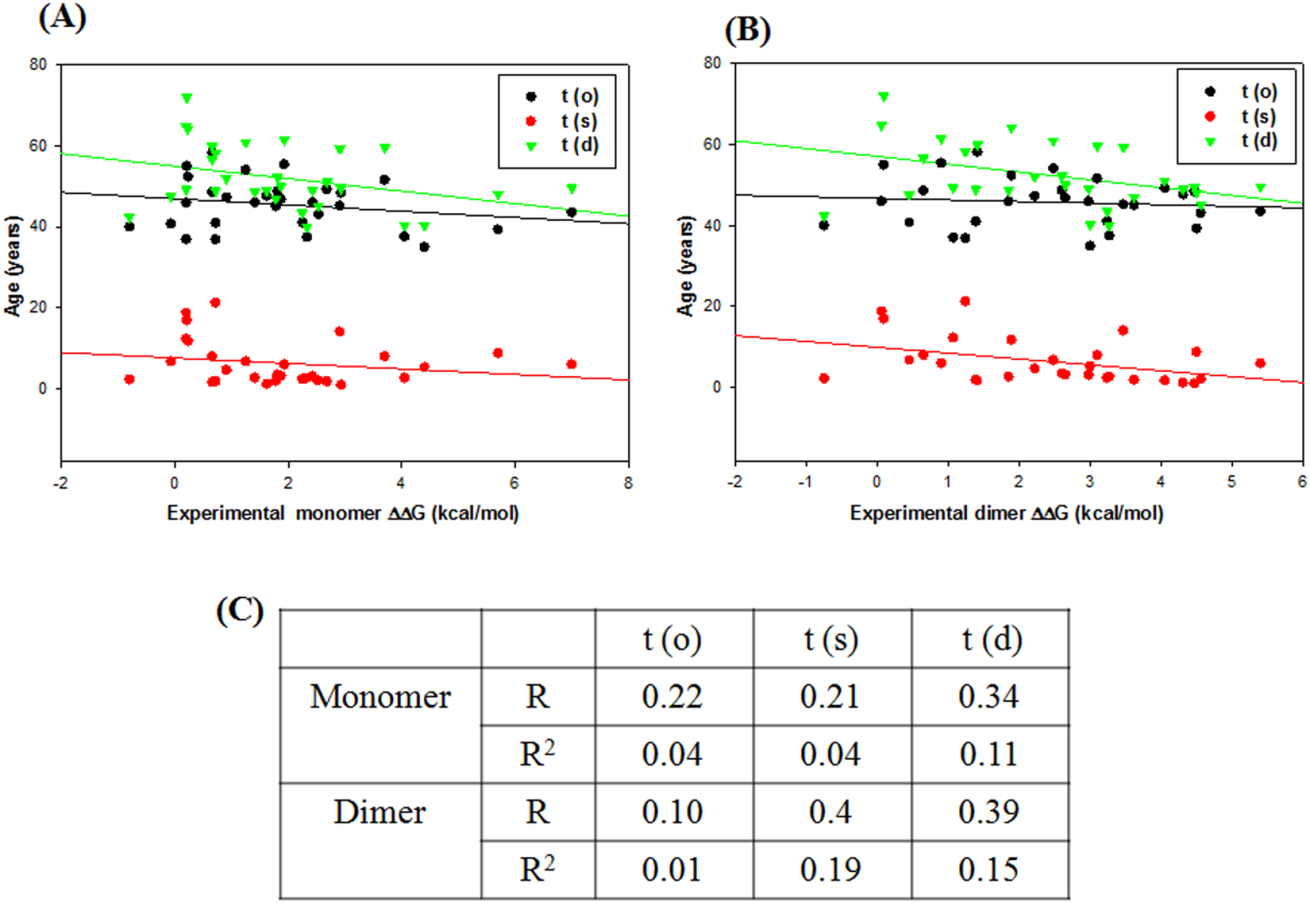
Correlation between patient’s disease onset, t(o), survival time, t(s), and age of death, t(d) (in years) of ALS patients carrying 30 SOD1 variants vs. the corresponding experimental monomer (**A**) and dimer (**B**) stability changes (kcal/mol). (**C**) Table lists linear regression coefficient, R and R^2^ value for correlation.

Patient data for age of onset, t(o), survival time, t(s), and age of death, t(d) = t(o) + t(s) were collected, analyzed and correlated against experimental and computed stabilities (Table S10). Figure 5 shows the correlation between disease phenotypes [t(o), t(s) and t(d)] and experimental ΔΔG of dimeric SOD1. Notably, disease duration, t(s) and disease death, t(d) correlates significantly with the experimentally measured stability change of the dimer (R = 0.4). However, disease onset, t(o) did not correlate with stability changes (R = 0.1), as also found previously^37, 38^. However, if we correlate patient data with monomer stability changes, we find that only t(d) correlates well with R = 0.34.

**Figure 6:**
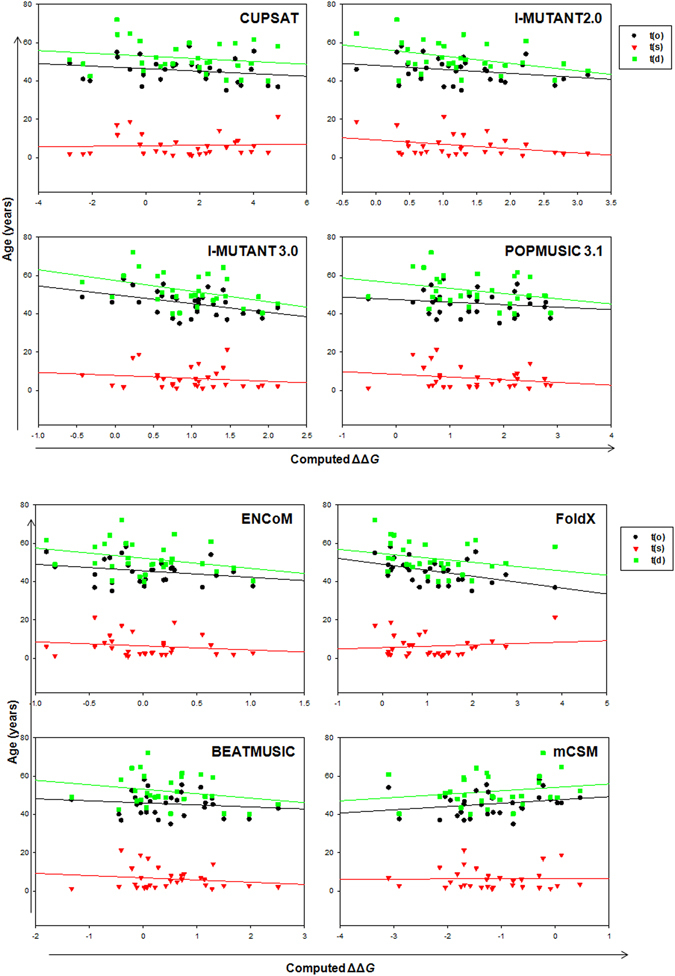
Correlation between predicted stability changes for 30 mutations in ALS with patients data, t(o), t(s) and t(d), using real holodimer of SOD1 structure, 2C9V.

Similarly, Fig. 6 shows the correlation between disease phenotypes [t(o), t(s) and t(d)] and predicted ΔΔ*G* for the representative and highest-resolution structure, 2C9V. The overall agreement with patient data was in several cases quite good (Table 2). In case of t(o), I-Mutant 3.0 and FoldX with R = 0.45 and even rest of the methods (R = > 0.15) correlate much better than experimental data (R = 0.1). For t(d), ENCoM (R = 0.47) and I-mutant 3.0/2.0 (R = 0.3) correlates well with experimental value (R = 0.4), while none of the methods shows good correlation in case of t(s), except I-mutant 3.0 (R = 0.33 as compared to R = 0.4). This holodimer structure (2C9V), on the average, gives the largest correlation coefficients mainly due to I-mutant 2.0 and I-mutant 3.0 (Table 2 and Supplementary Figure S6). The overall trend for the methods showed the following correlation: BeatMusic (R = 0.12) < CUPSAT (R = 0.14) < mCSM (R = 0.15) < PoPMuSiC 3.1 (R = 0.17) < ENCoM (R = 0.23) < I-Mutant 2 (R = 0.24) < FoldX (R = 0.25) < I-Mutant 3 (R = 0.28). Thus, by using different stability predictors, we were able to show, for the first time, that the methods are generally structure sensitive and correlate satisfactorily with ALS pathogenicity.

**Table 2:** Overall performance of stability calculators for SOD1 mutants in relation to patient’s data (Correlation coefficient, R value).

Methods		2C9V	1HL4	2XJK	4BCZ	Average
CUPSAT	t (o)	0.22	0.17	0.21	0.04	0.16
	t (s)	0.05	0.01	0.06	0.27	0.1
	t (d)	0.18	0.07	0.23	0.13	0.15
I-Mutant 2.0	t (o)	0.27	0.26	0.24	0.11	0.22
	t (s)	0.33	0.30	0.25	0.05	0.23
	t (d)	0.38	0.35	0.3	0.01	0.26
I-Mutant 3.0	t (o)	0.44	0.43	0.35	0.30	0.38
	t (s)	0.16	0.17	0.14	0.04	0.13
	t (d)	0.40	0.40	0.32	0.2	0.33
PoPMuSiC 3.1	t (o)	0.18	0.24	0.03	0.14	0.15
	t (s)	0.22	0.20	0.02	0.03	0.12
	t (d)	0.29	0.31	0.23	0.14	0.24
ENCoM	t (o)	0.24	0.31	0.09	0.15	0.2
	t (s)	0.17	0.19	0.14	0.31	0.2
	t (d)	0.30	0.36	0.47	0.08	0.3
FoldX	t (o)	0.46	0.44	0.40	0.28	0.4
	t (s)	0.12	0.09	0.11	0.31	0.16
	t (d)	0.25	0.25	0.20	0.03	0.18
BeatMusic	t (o)	0.14	0.11		0.03	0.09
	t (s)	0.16	0.13		0.03	0.1
	t (d)	0.23	0.18		0.04	0.15
mCSM	t (o)	0.24	0.32	0.26	0.21	0.26
	t (s)	0.006	0.03	0.03	0.02	0.02
	t (d)	0.19	0.23	0.20	0.04	0.16

## Conclusions

Protein stability plays a major role in biotechnology, pharmaceutical and food industries and in many diseases. The accuracy of the methods to predict the stability of protein variants are largely depend upon the structural data and experimental measurements as well as the complex physics of protein folding and stability. In this study, stability of human SOD1 variants have been computed using eight different methods and compared to known experimental stability data and patient’s phenotypes.

We report the validity of PoPMuSiC as a robust and reliable tool for the purpose of stability prediction. The main finding is that for the most accurate methods PoPMuSiC and FoldX, the different protein states do not significantly affect performance. It was also found that the most accurate methods are also less structure-sensitive. In addition, for several other stability predictors to get reliable estimation, choice of crystal structure is critical. Finally, this study also reports analysis of compiled patient data and experimental and computed protein stabilities for variants of human SOD1. Most importantly, patient’s disease duration t(s) correlates significantly with the experimentally measured and predicted stability change of the dimer SOD1. Thus, the present paper shows clearly the importance of protein stability in SOD1 pathogenicity.

## Methods

### Experimental data

The experimental data for the change in ΔΔ*G* (where Δ*G* is Gibbs free energy of folding), associated with single-point mutations, reported in the literature has been compiled and shown in Table S1. These data have been taken from Vassal *et al*.^39^ Nordlund and Oliveberg^40^, Lindberg *et al*.^28^, Stathopulos *et al*.^41^, and Bystrom *et al*.^42^. In total, 54 values of ΔΔ*G* for the apomonomer SOD1 mutants and 33 of the holodimer SOD1 mutants have been studied. The SOD1 mutations shown in Table S1 are grouped based on their positions in the structure as “β-barrel mutants” (B) and “metal binding mutants” (M). It has been observed that the methodological error in the experimental ΔΔ*G* is on the order of up to ~0.3 kcal/mol^28, 41^.

### Protein stability calculators

As in the previous work on SOD1^16^, eight stability predictors were used. Out of these eight methods, we have used CUPSAT^43^, POPMUSIC 2.1^44^, I-Mutant 2.0 and 3.0^45^. In addition to these, new methods, FoldX^46^, mCSM method^47^, PoPMuSiC 3.1 (https://soft.dezyme.com/), BeatMusic^48^ and ENCoM^49, 50^ were also used in this study. These eight methods used here are diverse in terms of their design philosophy and parameterization, but offer fast, web-based, quantitative estimates of stability effects of all possible mutations. Briefly, CUPSAT is based on atom potentials from chemical properties and empirically derived torsion potentials, whereas PoPMuSiC uses environment-specific statistical potentials based on observed substitution probabilities as developed by Topham *et al*.^51^. I-Mutant 2.0 and I-Mutant 3.0 are based on support vector machines that consider mainly amino acid substitution and structural environment, trained on experimental data points from the Protherm data base^52^. mCSM method is based on graph-based signatures using the atom distances to construct environments that are subsequently been trained on the experimental data. BeatMusic (http://babylone.ulb.ac.be/BeatMusic) relies on a set of statistical potentials derived from known protein structures, and combines the effect of the mutation on the strength of the interactions at the interface and on the overall stability of the complex. ENCoM is a coarse-grained normal mode analysis method recently introduced to predict the effect of mutations on protein dynamics and stability resulting from vibrational entropy changes. ENCoM employs a potential energy function that includes a pair-wise atom-type nonbonded interaction term and thus makes it unique in that it considers the nature of amino acids. While existing methods are based on machine learning or enthalpic considerations, ENCoM is based on entropic considerations^50^.

### Protein structures and structural sensitivity

As shown recently, the use of different crystal structures^16, 17^ or MD configurations^35^ can have very large impact on computed protein stabilities. Thus, structural sensitivity is an important parameter for protein property calculators. In the present work, four different crystal structures, 2C9V^53^, 1HL4^54^, 2XJK^55^, and 4BCZ^56^ of human SOD1were applied systematically to assess the accuracy of all eight methods. Properties of these four crystal structures are summarized in Table S11. Their crystallographic resolution values range from 1.07 (2C9V) to 1.93 (4BCZ), and pH values range from acidic to neutral. The root mean square deviation among the structures are provided in Table S11 and schematically represented in Supplementary Figure S7 in which the residues are color coded according to their RMSD values. 2C9V represents the physiologically relevant holodimer structure of high resolution with only one dimer. 1HL4 represents a non-metallated (apo) dimer form of the enzyme with two dimers in the structure. 2XJK is representative of a functional monomer structure with no other molecules in the unit cell. 4BCZ represents structure of apomonomer loopless SOD1 in which 44 residues (loops IV and VII) are removed and substituted with short Gly-Ala-Gly linkers. The resulting apoSOD1 barrel still has its structure intact. Thus, the four structures that have been chosen resemble commonly encountered heterogeneity in high resolution structures due to missing residues, pH, and crystal space group. The structural sensitivity was evaluated by computing the average ΔΔ*G* obtained for a mutation across all four crystal structures, then calculating the standard deviation from this average for each residue, and finally averaging over all computed data points.

### Analysis of predictor performance

The performance of the eight stability predictors was evaluated independently against both the apomonomer data set (54 data points) and the holodimer data set (33 data points). Results with all four structures were compared with the experimental data and were discussed on the basis of four important quality metrics. The correlation coefficient, R^2^ from a linear regression analysis describes the ability of a method to provide the overall trend in the data set; MSE, the mean signed error provides the systematic error and thus estimates the bias of the method towards destabilization or stabilization; and MAE, the mean absolute error describes the overall numerical accuracy of the method compared to experimental data. For a method, the structural robustness was computed as the standard deviation from the average of ΔΔ*G* values obtained for all structures.

A BA plot was used to analyze the agreement between the experimental and computed stability values. The BA test is a statistically robust method of accessing reliability and agreement between measurements. These plots quantify bias and provide a 95% confidence limits for the bias^57–59^. BA plots were constructed using Prism 6.

### Collection and analysis of patient data

SOD1 variants causing ALS and their associated patient data were collected from the compiled data from the ALS online genetics database^60^ and from Wang *et al*.^38^. Patient data were analyzed for age of onset, t(o); survival time, t(s); and age of death, t(d) = t(o) + t(s). These patient data were correlated against experimental and computed stabilities. Correlations were carried out linearly and subjected to regression analysis. R^2^ values will provide the overall trend in the data set.

## Electronic supplementary material

Supplementary Information
