# Supplementary material for: Computing disease-linked SOD1 mutations: deciphering protein stability and patient-phenotype relations
Source: Sci Rep. 2017 Jul 5;7:4678. doi: 10.1038/s41598-017-04950-9 (PMC5498623; doi:10.1038/s41598-017-04950-9)
Supplement: Supplementary file 1 — Supplementary Information [file 41598_2017_4950_MOESM1_ESM.pdf]

Supplementary Materials

Running Title: Stability prediction of SOD1 patient-phenotype pathogenesis

Computing disease-linked SOD1 mutations: deciphering protein stability and patient-phenotype relations

Vijay Kumar[1], Safikur Rahman[2], Hani Choudhry[3], Mazin A. Zamzami[4], Mohammad Sarwar Jamal[5], Asimul Islam[1], Faizan Ahmad[1] and Md. Imtaiyaz Hassan[1,*]

[1]Centre for Interdisciplinary Research in Basic Sciences, Jamia Millia Islamia, New Delhi – 110025, India

[2]Department of Medical Biotechnology, Yeungnam University, Gyeongsan, 712-749, South Korea

[3]Department of Biochemistry, Cancer Metabolism and Epigenetic Unit, Faculty of Science, Center of Innovation in Personalized Medicine, Cancer and Mutagenesis Unit, King Fahd Center for Medical Research, King Abdulaziz University, Jeddah, Saudi Arabia

[4]Department of Biochemistry, Cancer Metabolism and Epigenetic Unit, Faculty of Science, Cancer and Mutagenesis Unit, King Fahd Center for Medical Research, King Abdulaziz University, Jeddah, Saudi Arabia.

[5]King Fahd Medical Research Center, King Abdulaziz University, P.O. Box 80216 Jeddah, 21589, Kingdom of Saudi Arabia.

**To whom correspondence should be addressed:*

Md. Imtaiyaz Hassan, Ph.D.
Centre for Interdisciplinary Research in Basic Sciences,
Jamia Millia Islamia, New Delhi-110025, India
E-mail: mihassan@jmi.ac.in

Table S1. Effects of missense mutations on the stability of human SOD1

Mutation	ΔΔG(m)	ΔΔG(d)	Class	Reference
A4V	1.62	4.31	B	[1]
I18V	0.37		B	[2]
V29A	2.81		B	[2]
V31A	1.39		B	[2]
I35V	0.66		B	[2]
L38A	2.95		B	[2]
L38V	2.25	3.24	B	[3]
G41D	2.91	3.47	B	[1]
G41S	2.93	4.47	B	[1]
H43R	2.68	4.05	B	[1]
F45A	2.07		B	[2]
H46R	-0.81	-0.48	M	[1]
V47A	1.45		B	[2]
F64A	-0.2		B	[2]
D76V	0.2	0.06	M	[3]
D76Y	0.21	0.09	M	[3]
V81A	0.01		B	[2]
L84A	1.85		M	[2]
L84V	1.87	2.65	M	[1]
G85R	1.93	0.9	M	[1]
N86D	-0.05	0.94	B	[3]
N86K	0.66	1.41	B	[3]
N86S	-0.07	0.45	B	[3]
V87A	1.61		B	[2]
D90A	0.66	0.65	B	[1]
D90V	1.41	1.85	B	[3]
G93A	2.43	2.98	B	[1]
G93S	3.7	3.1	B	[4]
G93R	4.4	3	B	[4]
G93D	5.7	4.5	B	[4]
G93V	7	5.4	B	[4]
V97A	2.9		B	[2]
E100G	0.91	2.22	B	[1]
D101G	0.72	1.39	B	[3]
D101N	-0.8	-0.75	B	[3]
I104A	1.53		B	[2]
I104F	0.72	1.24	B	[1]
S105L	1.81	2.6	B	[3]
L106V	1.78	3.62	B	[1]
L106A	3.71		B	[2]
C111A	-0.42		B	[2]
I112A	1.25		B	[2]
I113T	1.25	2.48	B	[1]
G114A	2.33	3.27	B	[3]
L117A	1.78		B	[2]

V119A	2.55		B	[2]
N139D	0.01	-0.36	B	[3]
N139K	0.06	0.24	B	[3]
L144A	-0.13		B	[2]
L144S	0.2	1.07	B	[3]
L144F	0.23	1.89	B	[1]
V148G	2.53	4.56	B	[1]
I149A	4.05		B	[2]
I149V	0.27		B	[2]

Data in columns are from left: wild-type, position and mutant residue, free energy change for apo-monomer and holodimer in kcal/mol, ΔΔG(m) and ΔΔG(d), mutations class (β-sheet, B; metal-region, M; disulfide/cystine region, D)

Table S2. Experimental monomer and dimer stability changes and computed stability changes (kcal/mol) for 2C9V.

Mutations	ΔΔG(exp) (monomer)	ΔΔG(exp) (dimer)	CUPSAT	IMUTANT 2.0	IMUTANT 3.0	POPMUSIC 3.1	ENCoM	FoldX	BEATM USIC	mCSM
**A4V**	1.62	4.31	1.01	1.08	0.8	-0.52	-0.82	0.53	-1.33	-0.09
**I18V**	0.37		2.25	0.79	1.09	1.53	0.21	0.28	0.28	-1.57
**V29A**	2.81		4.96	1.37	1.88	2.56	0.56	1.22	0.92	-2.53
**V31A**	1.39		4.37	2.05	2.08	3.52	0.43	1.45	1.65	-2.48
**I35V**	0.66		2.7	0.45	0.88	1.47	0.19	0.48	0.1	-1.8
**L38A**	2.95		2.91	2.34	2.29	2.92	0.65	1.74	1.24	-2.62
**L38V**	2.25	3.24	2.24	0.67	1.07	1.35	0.02	1.78	0.06	-1.75
**G41S**	2.93	4.47	1.71	2.18	1.14	2.47	-0.14	1.3	1.28	-1.17
**G41D**	2.91	3.47	2.74	1.65	1.09	2.49	-0.1	0.94	1.3	-1.7
**H43R**	2.68	4.05	-2.84	1.33	0.61	1.36	0.17	1.15	0.07	-2.04
**F45A**	2.07		7.04	1.28	1.11	3.94	1.1	2.64	1.96	-2.87
**H46R**	-0.81	-0.48	-3.89	0.74	0.66	1.04	-0.55	2.9	-0.23	-1.91
**V47A**	1.45		5.52	1.19	1.3	2.91	0.57	1.2	1.2	-2.98
**F64A**	-0.2		2.76	2.57	2.14	2.75	1.22	1.27	1.07	-3.29
**D76Y**	0.21	0.09	-1.08	0.31	0.23	0.65	-0.2	-0.17	0.09	-0.22
**D76V**	0.2	0.06	-0.59	-0.29	0.31	0.31	0.29	0.18	-0.05	0.11
**V81A**	0.01		7.08	1.64	1.59	2.12	0.93	0.87	0.61	-2.49
**L84A**	1.85		4.48	1.85	1.79	2.9	1.02	1.22	1.19	-2.74
**L84V**	1.87	2.65	2.38	0.75	1.04	1.5	0.26	1.05	0.13	-1.68
**G85R**	1.93	0.9	4.03	0.7	0.63	2.25	-0.9	2.07	0.72	-1.27
**N86S**	-0.07	0.45	0.55	1.7	0.55	0.81	0.19	0.66	-0.04	-1.03
**N86D**	-0.05	0.94	-1.49	0.87	0.29	1.19	0.11	0.88	0.25	-0.91
**N86K**	0.66	1.41	1.62	0.38	0.11	0.88	-0.16	0.17	0.02	-0.29
**V87A**	1.61		2.33	1.82	1.62	2.38	0.58	0.9	0.83	-2.13
**D90V**	1.41	1.85	0.71	0.58	-0.04	0.68	0.08	0.58	0.42	0.03
**D90A**	0.66	0.65	1.12	0.47	-0.43	0.81	0.19	0.46	0.52	-0.3
**G93V**	7	5.4	2.29	0.48	1.05	2.76	-0.45	2.74	1.14	-0.63
**G93A**	2.43	2.98	3.93	1.62	1.44	2.78	0.09	1.46	1.16	-0.79
**G93R**	4.4	3	3	1.27	0.84	1.92	-0.29	1.98	0.51	-0.78
**G93D**	5.7	4.5	3.43	1.92	1.32	2.25	-0.29	2.44	0.76	-1.82
**G93S**	3.7	3.1	3.33	0.93	0.55	2.2	-0.36	1.88	0.71	-1.24
**V97A**	2.9		2.85	1.59	1.64	2.6	0.34	1.08	0.99	-1.7
**E100G**	0.91	2.22	1.94	1.26	1.09	0.74	0.27	0.21	0.63	-1.95
**D101G**	0.72	1.39	-2.34	2.79	1.87	1.5	0.21	1.68	0.22	-1.15
**D101N**	-0.8	-0.75	-2.08	1.86	1.67	0.61	-0.03	1	-0.45	-1.26
**I104A**	1.53		0.42	2.25	2.4	2.91	0.73	1.09	1.24	-2.91
**I104F**	0.72	1.24	4.92	1.01	1.46	0.75	-0.45	3.84	-0.41	-1.69
**S105L**	1.81	2.6	0.38	0.98	0.75	1.78	-0.14	0.12	-0.15	0.46
**L106V**	1.78	3.62	2	1.19	1.28	1	0.84	1.35	-0.12	-1.37
**L106A**	3.71		1.12	2.47	2.25	2.17	1.56	1.63	0.8	-2.32
**C111A**	-0.42		-3.29	0.48	1.18	1.48	0.07	0.02	0.15	-1.78
**I112A**	1.25		3.86	2.5	1.98	2.27	0.9	1.35	0.72	-3.21
**I113T**	1.25	2.48	-0.22	2.23	1.21	1.51	0.63	0.58	1.07	-3.1
**G114A**	2.33	3.27	4.56	0.34	0.75	2.2	0.01	1.24	1.97	-1.58
**L117A**	1.78		4.24	1.84	1.38	3.41	0.67	1.56	1.56	-3.07
**V119A**	2.55		2.56	0.78	0.93	3.58	0.5	1.32	1.68	-2.61
**N139K**	0.06	0.24	0.28	0.75	0.24	1.4	0.05	0.4	0.6	1.02

**N139D**	0.01	-0.36	2.28	0.26	-0.05	1	0.02	0.86	0.29	-1.92
**L144F**	0.23	1.89	-1.06	1.3	1.41	0.51	-0.31	0.25	-0.21	-1.47
**L144S**	0.2	1.07	-0.15	1.15	0.99	1.2	0.55	0.82	0.28	-2.15
**L144A**	-0.13		-0.65	0.75	0.61	1.43	0.8	0.73	0.44	-2.16
**V148G**	2.53	4.56	-0.09	3.15	2.12	2.11	0.68	0.12	2.51	-0.61
**I149A**	4.05		3.55	2.66	1.92	2.87	1.02	1.47	1.5	-2.9
**I149V**	0.27		2.5	0.62	0.64	1.06	0.34	0.36	-0.38	-1.68

Table S3. Experimental monomer and dimer stability changes and computed stability changes (kcal/mol) for 1HL4.

Mutations	ΔΔG(exp) (monomer)	ΔΔG(exp) (dimer)	CUPSAT	IMUTANT 2.0	IMUTANT 3.0	POPMUSIC 3.1	ENCOM	FOLDX	BEATMU SIC	mCSM
**A4V**	1.62	4.31	2.24	1.09	0.81	-0.49	-1.16	0.44	-1.31	-0.1
**I18V**	0.37		2.54	0.69	1.07	1.38	0.56	0.27	0.07	-1.38
**V29A**	2.81		5.16	1.69	1.96	2.65	0.56	1.36	1	-2.4
**V31A**	1.39		5.04	2.05	2.08	3.39	0.44	0.66	1.53	-1.59
**I35V**	0.66		2.36	0.43	0.78	1.39	0.27	0.32	0.12	-1.61
**L38A**	2.95		4.38	2.3	2.26	3.02	0.48	1.42	1.3	-2.65
**L38V**	2.25	3.24	2.41	0.64	1.06	1.54	-0.02	1.73	0.15	-1.76
**G41S**	2.93	4.47	2.07	2.36	1.19	2.32	-0.1	1.28	1.38	-1.01
**G41D**	2.91	3.47	2.51	1.76	1.09	2.35	-0.07	0.71	1.29	-1.43
**H43R**	2.68	4.05	-4.33	1.39	0.59	1.61	0.18	1.68	0.08	-1.99
**F45A**	2.07		7.3	1.39	1.28	3.9	1.17	2.13	2.01	-2.8
**H46R**	-0.81	-0.48	-3.67	0.74	0.65	1.13	-0.24	1.75	-0.19	-1.9
**V47A**	1.45		6.57	1.05	1.27	2.98	0.4	1.16	1.44	-2.69
**F64A**	-0.2		2.75	2.57	2.14	2.8	1.28	0.93	1.02	-3.14
**D76Y**	0.21	0.09	-0.92	0.56	0.32	0.88	-0.31	0.05	0.71	-0.31
**D76V**	0.2	0.06	-0.32	-0.03	0.42	0.25	0.29	0.28	-0.03	-0.03
**V81A**	0.01		4.8	1.56	1.56	1.93	0.83	0.74	0.59	-2.49
**L84A**	1.85		4.54	1.85	1.79	2.72	1.04	1.21	1.11	-2.66
**L84V**	1.87	2.65	3.21	0.74	1.04	1.31	0.21	0.89	0.03	-1.62
**G85R**	1.93	0.9	6.96	0.76	0.63	2.01	-0.92	1.79	0.74	-1.27
**N86S**	-0.07	0.45	-0.95	1.59	0.51	0.66	-0.1	0.49	-0.15	-1.05
**N86D**	-0.05	0.94	-2.21	0.79	0.28	0.96	-0.28	0.79	0.17	-0.97
**N86K**	0.66	1.41	-0.09	0.34	0.1	0.74	-0.8	-0.06	0.01	-0.29
**V87A**	1.61		3.62	1.89	1.63	2.44	0.56	0.93	0.85	-1.99
**D90V**	1.41	1.85	1.47	0.65	0	0.83	0.05	0.95	0.5	0.07
**D90A**	0.66	0.65	1.95	0.5	-0.42	0.93	0.18	0.82	0.56	-0.27
**G93V**	7	5.4	1.02	0.23	0.92	2.7	-0.46	2.19	1.09	-0.71
**G93A**	2.43	2.98	2.17	1.36	1.28	2.66	0.1	1.15	1.12	-0.9
**G93R**	4.4	3	1.07	1.19	0.72	1.97	-0.23	1.24	0.61	-0.88
**G93D**	5.7	4.5	0.42	1.88	1.24	2.24	-0.31	1.9	0.82	-2.0
**G93S**	3.7	3.1	2.76	0.71	0.36	2.14	-0.35	1.24	0.64	-1.36
**V97A**	2.9		6.35	1.53	1.67	2.55	0.33	1.13	0.83	-1.69
**E100G**	0.91	2.22	4.18	1.19	1	0.74	0.41	0.35	0.65	-0.74
**D101G**	0.72	1.39	-1.22	2.62	1.8	1.66	0.22	1.78	0.26	-0.95
**D101N**	-0.8	-0.75	0.12	1.8	1.65	0.79	-0.02	1.13	-0.37	-0.88
**I104A**	1.53		2.56	2.26	2.28	2.94	0.79	1.13	1	-2.62
**I104F**	0.72	1.24	4.02	1.07	1.4	0.85	-0.82	3.5	-0.46	-1.6
**S105L**	1.81	2.6	-2.58	0.92	0.72	0.91	0.06	0.63	-0.25	-0.35
**L106V**	1.78	3.62	1.95	1.18	1.27	1.08	0.54	0.96	-0.06	-1.44
**L106A**	3.71		5.37	2.46	2.25	2.29	1.49	1.56	0.62	-2.32
**C111A**	-0.42		-4.89	0.33	1.22	1.36	0.1	-0.06	0.37	-1.48
**I112A**	1.25		7.19	2.35	1.91	2.38	0.66	1.32	0.88	-2.3
**I113T**	1.25	2.48	2.11	2.27	1.28	1.71	0.31	0.92	1.17	-1.86
**G114A**	2.33	3.27	5.21	0.51	0.86	2.27	0.02	1.39	2.18	-1.17
**L117A**	1.78		5.05	1.71	1.3	3.29	0.82	1.53	1.49	-2.98
**V119A**	2.55		4.25	0.84	1.02	3.64	0.5	0.99	1.75	-2.55
**N139K**	0.06	0.24	0.9	0.76	0.24	1.65	-0.47	0.33	0.79	0.62
**N139D**	0.01	-0.36	1.08	0.26	-0.04	1	-0.38	0.85	0.23	-1.79

**L144F**	0.23	1.89	-0.69	1.04	1.22	0.58	-0.46	0.31	-0.19	-1.59
**L144S**	0.2	1.07	0.64	0.98	0.88	1.3	0.46	0.76	0.37	-2.22
**L144A**	-0.13		0.32	0.62	0.58	1.47	0.74	0.47	0.53	-2.25
**V148G**	2.53	4.56	-0.65	3.38	2.21	2.26	0.79	0.17	2.89	-1.0

Table S4. Experimental monomer and dimer stability changes and computed stability changes (kcal/mol) for 2XJK.

Mutations	ΔΔG(exp) (monomer)	ΔΔG(exp) (dimer)	CUPSAT	IMUTANT 2.0	IMUTANT 3.0	POPMUSIC 3.1	ENCOM	FOLDX	mCSM
**A4V**	1.62	4.31	-3.8	-0.94	-0.61	-0.36	-0.68	0.39	0.14
**I18V**	0.37		-2.56	-0.62	-1.06	1.24	-0.1	0.32	-1.43
**V29A**	2.81		-5.24	-1.64	-1.78	2.65	0.58	1.22	-2.49
**V31A**	1.39		-4.34	-2.09	-1.95	3.14	0.27	1.46	-1.76
**I35V**	0.66		-2.41	-0.27	-0.82	1.38	0.19	0.5	-1.76
**L38A**	2.95		-3.38	-2.35	-2.3	2.8	0.61	1.52	-2.74
**L38V**	2.25	3.24	-2.81	-0.68	-1.07	1.26	-0.02	1.81	-1.82
**G41S**	2.93	4.47	-6.51	-1.61	-1.05	1.95	-0.01	1.18	-1.05
**G41D**	2.91	3.47	-7.06	-2.15	-1.1	2.22	-0.08	0.65	-1.52
**H43R**	2.68	4.05	1.88	-1.34	-0.61	1.51	-0.15	1.94	-1.98
**F45A**	2.07		-6.7	-1.3	-1.17	4.02	1.16	2.58	-3.07
**H46R**	-0.81	-0.48	5.13	-0.73	-0.61	1.13	1.26	0.94	-1.93
**V47A**	1.45		-2.83	-1.33	-1.28	3.32	0.49	1.28	-2.98
**F64A**	-0.2		-2.24	-2.41	-2.1	3.01	1.39	1.45	-3.26
**D76Y**	0.21	0.09	0.89	0.16	-0.36	0.67	-0.67	0	-0.24
**D76V**	0.2	0.06	1.07	-0.39	-0.25	0.2	-0.06	0	-0.07
**V81A**	0.01		-4.24	-1.65	-1.65	2.02	0.93	0.5	-2.19
**L84A**	1.85		-3.87	-1.85	-1.79	2.8	1.24	1.37	-2.75
**L84V**	1.87	2.65	-1.7	-0.74	-1.04	1.39	0.18	1.37	-1.71
**G85R**	1.93	0.9	-3.95	-0.7	-0.63	2.32	-0.76	1.61	-1.23
**N86S**	-0.07	0.45	-2.36	-0.36	-0.09	0.52	0.24	0.45	-1.01
**N86D**	-0.05	0.94	-1.54	-1.68	-0.54	1.1	0.12	0.89	-0.84
**N86K**	0.66	1.41	1.67	-0.86	-0.29	0.85			-0.26
**V87A**	1.61		-3.36	-1.81	-1.61	2.34	0.65	0.93	-2.1
**D90V**	1.41	1.85	-2.33	-0.39	0.5	0.73	0.22	0.67	0.13
**D90A**	0.66	0.65	-1.22	-0.43	0.15	0.7	0.07	0.57	-0.21
**G93V**	7	5.4	-3.24	-0.93	-0.55	2.93	-0.34	3.01	-0.65
**G93A**	2.43	2.98	-1.76	-0.48	-1.05	2.83	0.1	1.57	-0.81
**G93R**	4.4	3	-2.53	-1.29	-0.84	2.06	-0.26	2.39	-0.78
**G93D**	5.7	4.5	-2.47	-1.93	-1.32	2.43	-0.27	2.81	-1.81
**G93S**	3.7	3.1	-3.03	-1.64	-1.44	2.47	-0.38	2.09	-1.24
**V97A**	2.9		-5.27	-1.82	-1.75	2.64	0.46	1.17	-1.71
**E100G**	0.91	2.22	-3.05	-1.92	-1.27	1.06	0.74	0.31	-0.56
**D101G**	0.72	1.39	-0.18	-3.07	-1.98	1.56	0.29	1.68	-0.85
**D101N**	-0.8	-0.75	-0.3	-2.01	-1.79	0.71	0.48	1.27	-0.82
**I104A**	1.53		-1.17	-2.16	-2.4	2.85	0.63	1.35	-2.25
**I104F**	0.72	1.24	-2.69	-0.91	-1.42	0.74	-0.65	4.72	-1.36
**S105L**	1.81	2.6	-1.38	-0.72	-0.61	0.93	-0.03	0.75	-0.64
**L106V**	1.78	3.62	-1.81	-1.06	-1.12	0.92	0.77	1.2	-1.5
**L106A**	3.71		-2.9	-2.49	-2.1	2.19	1.51	1.56	-2.44
**I112A**	1.25		-1.3	-2.81	-2.14	2.4	0.83	1.34	-2.28
**I113T**	1.25	2.48	-1.64	-2.47	-1.31	0.7	0.38	0.87	-1.49
**G114A**	2.33	3.27	-1.61	-0.37	-0.53	1.76	-0.08	1.13	-0.61
**L117A**	1.78		-2.79	-1.82	-1.39	3.13	0.69	1.53	-3.05
**V119A**	2.55		-3.63	-0.78	-0.84	3.63	0.49	1.32	-2.79
**N139K**	0.06	0.24	-1.66	-0.26	0.04	1.78	0.04	0.01	0.33
**N139D**	0.01	-0.36	0.49	-0.76	-0.24	1.05	0.01	0.6	-1.54
**L144F**	0.23	1.89	0.34	-0.81	-0.55	0.62	-0.35	0.41	-1.46

**L144S**	0.2	1.07	-1.67	-1.19	-0.88	1.23	0.56	0.77	-1.86
**L144A**	-0.13		0.27	-1.26	-1.25	1.56	0.8	0.71	-1.98
**V148G**	2.53	4.56	-2.01	-2.42	-1.81	0.52	0.12	0.14	-0.96
**I149A**	4.05		-2.71	-3.37	-2.13	2.61	0.99	1.62	-2.34
**I149V**	0.27		-1.67	-0.42	-0.56	1.09	0.2	0.39	-1.16

Table S5. Experimental monomer and dimer stability changes and computed stability changes (kcal/mol) for 4BCZ.

Mutations	ΔΔG$_{(exp)}$ (monomer)	ΔΔG$_{(exp)}$ (dimer)	CUPSAT	IMUTANT 2.0	I-MUTANT 3.0	POPMUSIC 3.1	ENCoM	FoldX	BEATMUSIC	mCSM
**A4V**	1.62	4.31	-0.39	-0.91	-0.68	-0.44	-0.96	0.3	-1.47	0.04
**I18V**	0.37		-2.38	-0.58	-1.02	1.37	0.14	0.27	0.03	-1.21
**V29A**	2.81		-5.99	-1.26	-1.7	2.6	0.52	1.15	1.05	-2.4
**V31A**	1.39		-7.38	-2.13	-1.9	3.47	0.49	1.45	1.71	-2.22
**I35V**	0.66		-2.22	-0.22	-0.72	1.44	0.2	0.48	0.16	-2.02
**L38A**	2.95		-2.97	-1.76	-1.79	2.3	0.82	1.59	0.81	-2.67
**L38V**	2.25	3.24	-0.81	-0.58	-0.88	1.21	0.1	1.65	0	-1.8
**G41S**	2.93	4.47	-1.35	-1.7	-0.86	2.43	-0.14	1.08	1.3	-1.22
**G41D**	2.91	3.47	-0.8	-2.45	-1	2.54	-0.11	0.47	1.39	-1.64
**H43R**	2.68	4.05	3.11	-1.25	-0.46	1.34	0.44	2.59	0.01	-1.82
**F45A**	2.07		-5.71	-1.86	-1.32	3.58	1.27	2.54	1.68	-2.66
**H46R**	-0.81	-0.48	2.02	-0.74	-0.48	0.56	0.27	-0.16	-0.24	-1.38
**V47A**	1.45		-4.29	-1.35	-1.39	2.06	0.58	0.99	0.46	-2.21
**L84A**	1.85		-0.29	-2.44	-2.05	2.84	1.51	1.32	1.19	-2.19
**L84V**	1.87	2.65	0.46	-0.87	-1.1	1.42	0.64	1.16	0.13	-1.31
**G85R**	1.93	0.9	0.24	-0.46	-0.67	2.05	-0.01	1.3	1.28	-0.73
**N86S**	-0.07	0.45	1.52	-0.46	-0.05	0.01	0.14	0.2	-0.06	-0.32
**N86D**	-0.05	0.94	0.33	-1.66	-0.43	0.32	-0.13	0.15	0.18	0.25
**N86K**	0.66	1.41	0.31	-1.28	-0.29	0.29			0.11	0.13
**V87A**	1.61		-2.5	-1.05	-1.27	2.32	0.64	0.85	0.78	-2.22
**D90V**	1.41	1.85	-1.9	-0.43	0.47	0.97	-0.06	1.07	0.47	0.03
**D90A**	0.66	0.65	-2.11	-0.55	0.07	0.98	0.06	0.68	0.48	-0.3
**G93V**	7	5.4	-3.86	-1.21	-0.42	2.68	-0.35	2.35	1.11	-0.66
**G93A**	2.43	2.98	-2.61	-0.7	-0.9	2.62	0.02	1.21	1.05	-0.84
**G93R**	4.4	3	-3.7	-1.37	-0.69	1.99	-0.33	1.46	0.57	-0.81
**G93D**	5.7	4.5	-1.98	-2.16	-1.19	2.22	-0.33	1.82	0.76	-1.85
**G93S**	3.7	3.1	-3.56	-1.59	-1.22	2.07	-0.53	1.61	0.64	-1.28
**V97A**	2.9		-4.89	-1.5	-1.58	2.61	0.24	1.15	0.97	-2.02
**E100G**	0.91	2.22	-1	-1.58	-1.02	0.79	0.65	0.35	0.7	-0.96
**D101G**	0.72	1.39	0.48	-2.77	-1.56	1.71	0.11	0.6	0.48	-0.86
**D101N**	-0.8	-0.75	-0.25	-1.77	-1.51	0.74	-0.03	-0.18	-0.22	-0.76
**I104A**	1.53		-1.7	-2.33	-2.11	2.18	0.7	0.91	0.69	-2.01
**I104F**	0.72	1.24	-4.09	-1.23	-1.34	0.51	-0.84	3.89	-0.56	-1.26
**S105L**	1.81	2.6	4.62	-0.36	-0.3	0.94	-0.11	0.35	-0.12	-0.79
**L106V**	1.78	3.62	-2.57	-1.31	-1.34	1.18	0.63	1.5	-0.1	-1.67
**L106A**	3.71		-4.21	-2.4	-2.3	2.31	1.51	1.46	0.81	-2.45
**I112A**	1.25		-5.96	-2.54	-1.85	2.2	0.75	1.25	0.55	-2.31
**I113T**	1.25	2.48	-6.58	-2.94	-1.56	1.06	0.95	0.38	0.19	-1.37
**G114A**	2.33	3.27	1.77	-0.46	-0.79	1.33	-0.01	1.3	1.02	-0.26
**L117A**	1.78		-4.37	-1.56	-1.26	3.14	0.72	1.71	1.35	-2.81
**V119A**	2.55		-2.85	-0.82	-0.92	3.1	0.41	1.43	1.33	-2.62
**L144F**	0.23	1.89	-0.38	-1.12	-0.69	0.23	0.15	-0.17	-0.25	-1.22
**L144S**	0.2	1.07	1.25	-1.36	-1.17	0.92	0.15	0.96	0.27	-1.25
**L144A**	-0.13		0.54	-1.74	-1.55	1.29	0.33	0.73	0.54	-1.52
**V148G**	2.53	4.56	-0.32	-3.06	-2.2	0.59	0.15	0.12	0.4	-0.57
**I149A**	4.05		-2.63	-2.61	-2.15	2.71	0.98	1.64	1.06	-2.31
**I149V**	0.27		-2.08	-0.2	-0.68	1.04	0.19	0.44	-0.19	-1.18

Table S6. Bland Altman analysis of mean differences between experimental and computed stability predictors

PDB	Stability Predictors	Bias	SD of Bias	Mean ± 1.96 SD
Dimer/Monomer		0.519091	0.983846	2.44; -1.40
2C9V	CUPSAT	0.228519	2.37008	4.87; -4.41
	I-Mutant 2.0	-0.276667	1.59319	2.85;-3.39
	I-Mutant 3.0	-0.464630	1.55982	2.84; -3.40
	PoPMuSiC 3.1	0.230185	1.38360	2.94; -2.48
	ENCoM	-1.33296	1.68868	1.98; -4.64
	FoldX	-0.453889	1.41646	2.32; -3.23
	BeatMusic	-0.969815	1.41701	1.81; -3.75
	mCSM	-3.22093	1.92398	0.55; -6.99
1HL4	CUPSAT	0.511154	2.91821	6.23; -5.21
	I-Mutant 2.0	-0.283077	1.61560	2.88; -3.45
	I-Mutant 3.0	-0.465192	1.57707	2.63; -3.56
	PoPMuSiC 3.1	0.241154	1.39334	2.97; -2.49
	ENCoM	-1.38808	1.67719	1.90; -4.67
	FoldX	-0.537115	1.40872	2.22; -3.30
	BeatMusic	-0.915000	1.45559	1.94; -3.77
	mCSM	-3.07423	1.85555	0.56; -6.71
2XJK	CUPSAT	-3.80075	3.21343	2.50; -10.10
	I-Mutant 2.0	-2.97340	1.95576	0.86; -6.81
	I-Mutant 3.0	-2.66113	1.78681	0.84; -6.16
	PoPMuSiC 3.1	0.170943	1.93553	3.96; -3.62
	ENCoM	-1.36154	1.75475	2.08; -4.80
	FoldX	-0.447692	1.34523	2.19; -3.08

	mCSM	-3.11321	1.91271	0.63; -6.86
4BCZ	CUPSAT	-3.61702	3.40870	3.06; -10.23
	I-Mutant 2.0	-3.24787	1.89115	0.46; -6.95
	I-Mutant 3.0	-2.93170	1.77633	0.55; -6.41
	PoPMuSiC 3.1	-0.173404	1.30507	2.38; -2.73
	ENCoM	-1.58239	1.67567	1.70; -4.87
	FoldX	-0.780652	1.33171	1.83; -3.39
	BeatMusic	-1.30787	1.38584	1.41; -4.02
	mCSM	-3.22298	1.93226	0.56; -7.01

Table S7. Overall performance of stability predictors for 33 SOD1 holo-dimer mutants using four different crystal structures

		Addiditivity Assumption[a]				
Methods	Metrics	2C9V	1HL4	2XJK	4BCZ	Average
CUPSAT	MAE	1.6	1.75	3.21	2.6	2.3
	MSE	1.08	1.14	3.73	3.19	2.28
	R	0.38	0.17	0.53	0.23	0.33
	R^2	0.144	0.03	0.28	0.05	0.13
I-Mutant 2.0	MAE	0.68	0.68	2.2	1.91	1.36
	MSE	0.99	1.00	3.26	3.75	2.25
	R	0.35	0.34	0.3	0.23	0.3
	R^2	0.12	0.11	0.09	0.05	0.09
I-Mutant 3.0	MAE	0.65	0.65	1.28	2.09	1.17
	MSE	1.28	1.31	2.93	3.26	2.19
	R	0.41	0.4	0.31	0.2	0.33
	R^2	0.17	0.16	0.1	0.04	0.12
PoPMuSiC 3.1	MAE	0.65	0.62	0.69	0.61	0.64
	MSE	0.76	0.75	0.85	1.21	0.89
	R	0.51	0.5	0.38	0.45	0.46
	R^2	0.26	0.25	0.14	0.2	0.21
ENCoM	MAE	1.13	1.21	1.21	1.27	1.21
	MSE	2.15	2.24	2.11	2.41	2.22
	R	0.001	0.1	0.25	0.16	0.13
	R^2	0	0.01	0.06	0.02	0.02
FoldX	MAE	0.8	0.73	0.73	0.66	0.73
	MSE	0.99	1.09	0.97	1.46	1.13
	R	0.18	0.19	0.32	0.37	0.26
	R^2	0.03	0.03	0.1	0.13	0.07
BeatMusic	MAE	0.77	0.81		0.96	0.85
	MSE	1.72	1.66		2.10	1.83
	R	0.41	0.37		0.25	0.34
	R^2	0.17	0.14		0.06	0.12
mCSM	MAE	2.23	2.15	2.05	2.13	2.14
	MSE	3.23	3.19	3.11	3.34	3.22
	R	0.11	0.16	0.16	0.25	0.17
	R^2	0.01	0.03	0.02	0.06	0.03

[a] Additivity assumption: Calculated and multiplied by 2

Table S8. Computed stability changes (kcal/mol), averaged over all four structures.

Mutations	CUPSAT	IMUT-2.0	IMUT-3.0	POPMUSIC 3.1	ENCoM	FoldX	BEATMUSIC	mCSM
**A4V**	-0.235	0.08	0.08	-0.452	-0.9	0.42	-1.37	-0.004
**I18V**	-0.04	0.07	0.02	1.38	0.2	0.3	0.13	-1.4
**V29A**	-0.28	0.04	0.09	2.61	0.55	1.24	1	-2.45
**V31A**	-0.57	-0.03	0.08	3.4	0.4	1.25	1.63	-2.01
**I35V**	0.1	0.09	0.03	1.42	0.21	0.45	0.13	-1.8
**L38A**	0.23	0.13	0.11	2.76	0.64	1.57	1.12	-2.67
**L38V**	0.26	0.01	0.045	1.34	0.02	1.74	0.07	-1.78
**G41D**	-0.65	-0.3	0.02	2.4	-0.09	0.7	1.33	-1.57
**G41S**	-1.02	0.3	0.1	2.3	-0.09	1.21	1.32	-1.11
**H43R**	-0.54	0.03	0.032	1.45	0.16	1.84	0.05	-1.96
**F45A**	0.48	-0.12	-0.02	3.86	1.17	2.47	1.88	-2.85
**H46R**	-0.1	0.002	0.05	0.96	0.18	1.36	-0.22	-1.78
**V47A**	1.24	-0.11	-0.02	2.82	0.52	1.16	1.03	-2.72
**F64A**	1.09	0.91	0.73	2.85	1.29	1.21	1.04	-3.23
**D76V**	0.05	-0.24	0.16	0.25	0.17	0.15	-0.04	0.001
**D76Y**	-0.37	0.34	0.06	0.73	-0.39	-0.04	0.4	-0.26
**V81A**	2.54	0.52	0.5	2.02	0.9	0.7	0.6	-2.4
**L84A**	2.42	0.92	0.9	2.78	1.08	1.25	1.14	-2.7
**L84V**	1.08	-0.03	-0.02	1.4	0.32	1.12	0.096	-1.58
**G85R**	1.82	0.07	-0.01	2.15	-0.65	1.7	0.91	-1.13
**N86D**	-1.23	-0.42	-0.1	0.9	-0.04	0.68	0.2	-0.62
**N86K**	0.87	-0.35	-0.09	0.7	-0.48	0.06	0.05	-0.18
**N86S**	-0.31	0.62	0.23	0.5	0.12	0.45	-0.08	-0.85
**V87A**	0.022	0.021	0.09	2.37	0.6	0.9	0.82	-2.11
**D90A**	-0.06	-0.002	-0.16	0.85	0.125	0.63	0.52	-0.27
**D90V**	-0.51	0.1	0.23	0.8	0.07	0.821	0.46	0.06
**G93A**	0.43	0.45	0.19	2.72	0.07	1.35	1.11	-0.84
**G93S**	-0.12	-0.4	-0.44	2.22	-0.4	1.7	0.66	-1.28
**G93R**	-0.54	-0.05	0.007	1.98	-0.27	1.76	0.56	-0.81
**G93D**	-0.15	-0.07	0.01	2.3	-0.3	2.24	0.78	-1.87
**G93V**	-0.94	-0.36	0.25	2.76	-0.4	2.57	1.11	-0.66
**V97A**	-0.24	-0.05	-0.005	2.6	0.34	1.14	0.93	-1.78
**E100G**	0.52	-0.26	-0.05	0.83	0.52	0.3	0.66	-1.05
**D101G**	-0.81	-0.1	0.03	1.6	0.21	1.44	0.32	-0.95
**D101N**	-0.62	-0.03	0.005	0.71	0.09	0.8	-0.34	-0.93
**I104A**	0.03	0.005	0.04	2.72	0.71	1.12	0.97	-2.44
**I104F**	0.54	-0.01	0.02	0.71	-0.68	3.98	-0.47	-1.48
**S105L**	0.26	0.2	0.14	1.14	-0.05	0.47	-0.17	-0.33
**L106V**	-0.1	0	0.02	1.04	0.69	1.25	-0.09	-1.49
**L106A**	-0.15	0.01	0.025	2.24	1.52	1.55	0.75	-2.38
**C111A**	-4.09	0.4	1.2	1.42	0.08	-0.02	0.26	-1.63
**I112A**	0.95	-0.12	-0.025	2.31	0.78	1.32	0.71	-2.5
**I113T**	-1.58	-0.23	-0.09	1.24	0.56	0.7	0.8	-1.95
**G114A**	2.48	0.005	0.07	1.89	-0.015	1.27	1.72	-0.9
**L117A**	0.53	0.04	0.007	3.24	0.73	1.58	1.46	-2.98
**V119A**	0.08	0.005	0.04	3.48	0.47	1.27	1.58	-2.64
**N139D**	1.28	-0.08	-0.11	1.01	-0.11	0.77	0.26	-1.75
**N139K**	-0.16	0.42	0.17	1.61	-0.13	0.24	0.7	0.65
**L144A**	0.12	-0.4	-0.4	1.43	0.66	0.66	0.5	-1.97
**L144S**	0.02	-0.1	-0.04	1.16	0.43	0.82	0.3	-1.87
**L144F**	-0.44	0.1	0.34	0.48	-0.24	0.2	-0.21	-1.4

**V148G**	-0.76	0.26	0.08	1.37	0.44	0.14	1.93	-0.78
**I149A**	0.38	-0.16	-0.11	2.74	0.98	1.53	1.34	-2.54
**I149V**	0.32	0.15	0.01	1.06	0.28	0.38	-0.33	-1.37

Table S9. Standard Deviations from average (kcal/mol) over all four structures (Structural Sensitivity).

Mutations	CUPSAT	IMUT-2.0	IMUT-3.0	POPMUS IC 3.1	FoldX	ENCoM	BEATM USIC	mCSM
A4V	2.26	1	0.72	0.06	0.82	0.18	0.07	0.1
I18V	2.43	0.67	1.1	0.1	0.02	0.23	0.1	0.12
V29A	5.34	1.5	1.84	0.03	0.08	0.02	0.05	0.05
V31A	5.4	2.08	2	0.15	0.34	0.08	0.07	0.35
I35V	2.43	0.35	0.8	0.04	0.07	0.03	0.02	0.14
L38A	3.45	2.2	2.2	0.28	0.12	0.12	0.22	0.04
L38V	2.18	0.65	1.02	0.12	0.07	0.05	0.06	0.03
G41D	3.95	2	1.07	0.12	0.18	0.02	0.045	0.1
G41S	3.44	1.96	1.06	0.2	0.09	0.05	0.043	0.08
H43R	3.11	1.32	0.57	0.11	0.52	0.21	0.03	0.08
F45A	6.7	1.47	1.22	0.17	0.2	0.06	0.14	0.14
H46R	3.84	0.74	0.6	0.24	1.1	0.29	0.02	0.23
V47A	4.84	1.23	1.31	0.47	0.1	0.07	0.42	0.31
F64A	2.35	2.35	2	0.11	0.21	0.07	0.02	0.07
D76V	0.73	0.16	0.3	0.04	0.11	0.17	0.01	0.08
D76Y	0.9	0.16	0.3	0.1	0.09	0.2	0.3	0.04
V81A	4.88	1.53	1.52	0.07	0.15	0.05	0.01	0.14
L84A	3.63	1.6	1.55	0.07	0.06	0.09	0.04	0.04
L84V	1.9	0.78	1.05	0.07	0.18	0.18	0.05	0.16
G85R	4.1	0.66	0.65	0.13	0.29	0.37	0.26	0.23
N86D	0.94	1.25	0.38	0.34	0.3	0.17	0.03	0.5
N86K	0.78	0.73	0.2	0.24	0.12	0.32	0.04	0.18
N86S	1.47	1.02	0.3	0.3	0.17	0.13	0.05	0.3
V87A	3	1.67	1.53	0.04	0.03	0.03	0.03	0.08
D90A	1.65	0.49	0.26	0.11	0.13	0.06	0.03	0.04
D90V	1.63	0.51	0.25	0.11	0.2	0.1	0.03	0.04
G93A	2.7	1.04	1.17	0.08	0.17	0.03	0.04	0.04
G93S	3.18	1.22	0.9	0.15	0.32	0.07	0.03	0.05
G93R	2.7	1.28	0.77	0.05	0.45	0.04	0.04	0.04
G93D	2.33	1.97	1.27	0.08	0.4	0.02	0.03	0.07
G93V	2.65	0.72	0.74	0.09	0.32	0.05	0.02	0.03
V97A	5	1.61	1.66	0.03	0.03	0.08	0.07	0.13
E100G	2.75	1.5	1.1	0.13	0.05	0.19	0.03	0.53
D101G	1.06	2.81	1.8	0.08	0.49	0.06	0.11	0.12
D101N	0.85	1.86	1.66	0.06	0.58	0.22	0.09	0.19
I104A	1.65	2.25	2.3	0.31	0.16	0.06	0.22	0.35
I104F	4	1.06	1.4	0.12	0.45	0.16	0.06	0.18
S105L	2.72	0.76	0.6	0.37	0.24	0.08	0.05	0.48
L106V	2.09	1.19	1.25	0.09	0.2	0.12	0.02	0.11
L106A	3.74	2.45	2.23	0.06	0.06	0.02	0.09	0.06
C111A	0.8	0.07	0.02	0.06	0.04	0.01	0.11	0.15
I112A	5	2.55	1.97	0.08	0.04	0.09	0.13	0.4
I113T	3.18	2.48	1.34	0.4	0.22	0.25	0.44	0.68
G114A	2.7	0.42	0.74	0.37	0.1	0.04	0.5	0.5
L117A	4.16	1.74	1.33	0.12	0.07	0.06	0.09	0.1
V119A	3.4	0.8	0.93	0.22	0.17	0.04	0.18	0.09
N139D	0.75	0.48	0.09	0.02	0.12	0.19	0.03	0.16
N139K	1.1	0.48	0.09	0.16	0.17	0.24	0.09	0.28
L144A	0.46	1.1	1.03	0.1	0.1	0.2	0.04	0.28

L144S	1.1	1.17	0.98	0.14	0.08	0.17	0.04	0.38
L144F	0.51	1.07	0.97	0.15	0.22	0.23	0.02	0.13
V148G	0.75	3.01	2.1	0.12	0.02	0.3	0.09	0.2
I149A	3.05	2.84	2	0.09	0.1	0.02	0.2	0.24
I149V	2.2	0.47	0.64	0.02	0.04	0.08	0.1	0.22

Table S10. SOD1 variants with experimentally known stabilities (kcal/mol) correlated against t(o), t(s), and t(d) (years).

Mutations	ΔΔG(exp) (monomer)	ΔΔG(exp) (dimer)	t(o)	t(s)	t(d)	Reference
**A4V**	1.62	4.31	47.7	1.2	48.9	[5]
**L38V**	2.25	3.24	41.1	2.4	43.5	[6]
**G41S**	2.93	4.47	48.4	1	49.4	[7]
**G41D**	2.91	3.47	45.2	14.1	59.3	[8]
**H43R**	2.68	4.05	49.3	1.8	51.1	[9]
**D76Y**	0.21	0.09	55	17	72	[10]
**D76V**	0.2	0.06	46	18.8	64.8	[11]
**L84V**	1.87	2.65	46.8	3.2	50	[12]
**G85R**	1.93	0.9	55.5	6	61.5	[13]
**N86S**	-0.07	0.45	40.8	6.8	47.6	[14]
**N86K**	0.66	1.41	58.2	1.7	59.9	[15]
**D90V**	1.41	1.85	46	2.7	48.7	[16]
**D90A**	0.66	0.65	48.6	8	56.6	[17]
**G93V**	7	5.4	43.6	6	49.6	[18]
**G93A**	2.43	2.98	46	3.1	49.1	[19]
**G93R**	4.4	3	35	5.3	40.3	[20]
**G93D**	5.7	4.5	39.3	8.8	48.1	[21]
**G93S**	3.7	3.1	51.6	8	59.6	[22]
**E100G**	0.91	2.22	47.3	4.7	52	[8]
**D101G**	0.72	1.39	41	1.9	48.9	[23]
**D101N**	-0.8	-0.75	40.1	2.3	42.4	[24]
**I104F**	0.72	1.24	36.9	21.3	58.2	[25]
**S105L**	1.81	2.6	48.7	3.5	52.2	[26]
**L106V**	1.78	3.62	45.1	1.9	47	[8]
**I113T**	1.25	2.48	54.1	6.8	60.9	[27]
**G114A**	2.33	3.27	37.5	2.7	40	[28]
**L144F**	0.23	1.89	52.4	11.8	64.2	[29]
**L144S**	0.2	1.07	37	12.3	49.3	[30]
**V148G**	2.53	4.56	43.1	2.1	45.2	[31]
**I149T**	4.05		37.6	2.7	40.3	[32]

Table S11. SOD1 structures used for computation of mutant stabilities

PDB	Stoichiometry	R (Å)	Space Group	pH	RMSD*	No. of residues
2C9V	Homo-2-mer	1.07	P 1211	4.75	-	306
1HL4	Homo-2-mer	1.82	C 121	8.0	0.35	306
2XJK	Monomer	1.45	P 212121	6.0	0.29	153
4BCZ	Monomer	1.93	P 65	4.0	11.16	110

*RMSD with respect to 2C9V

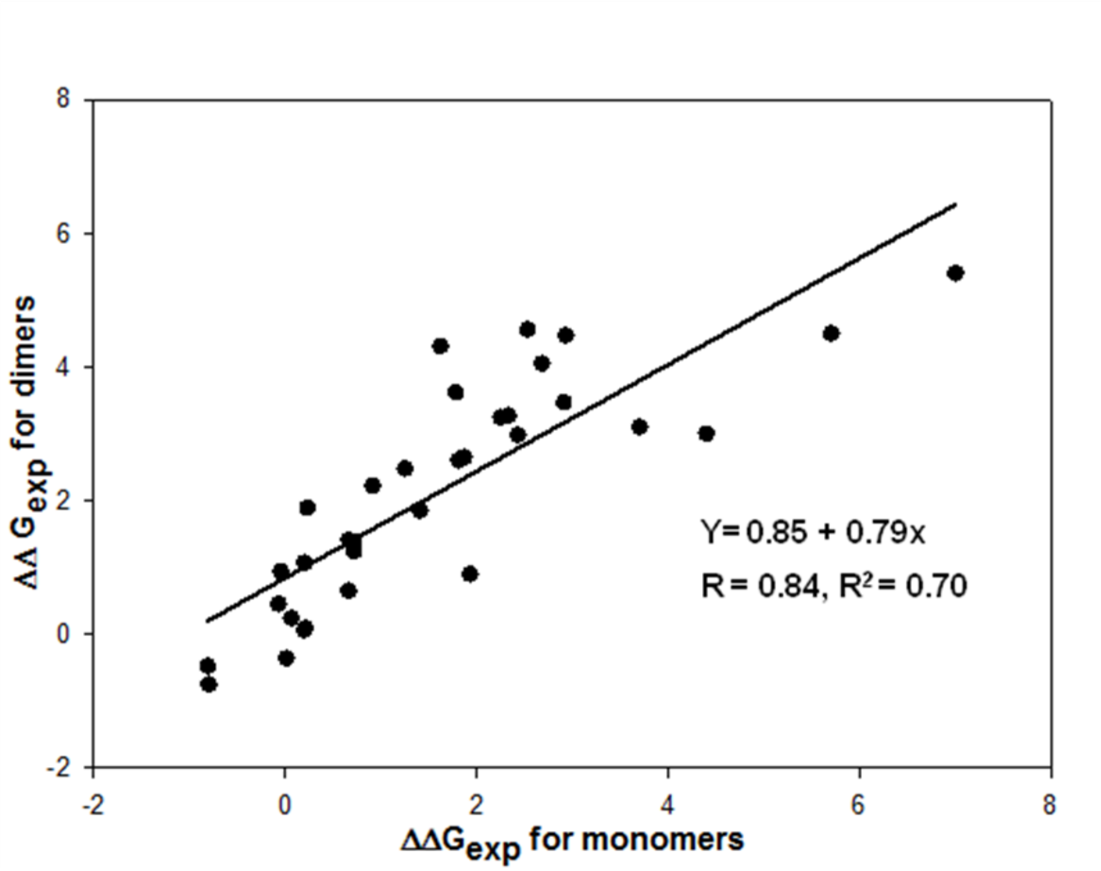

Figure S1: Correlation between experimental free energies (kcal/mol) of mutants for dimer and monomer SOD1.

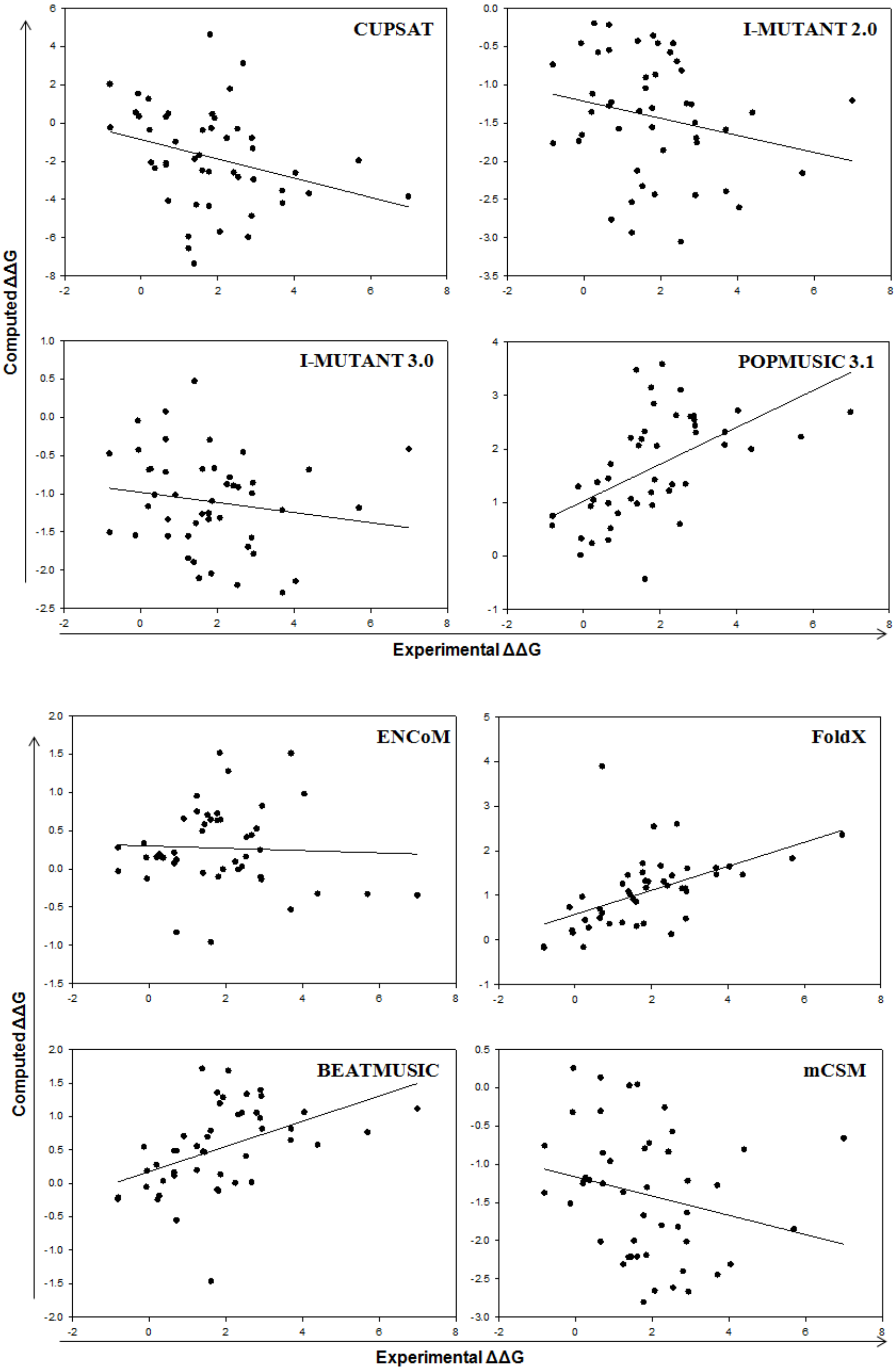

Figure S2. Correlation between experimental monomer and computed stability changes, ΔΔ*G* (kcal/mol) for 54 mutants using 4BCZ.

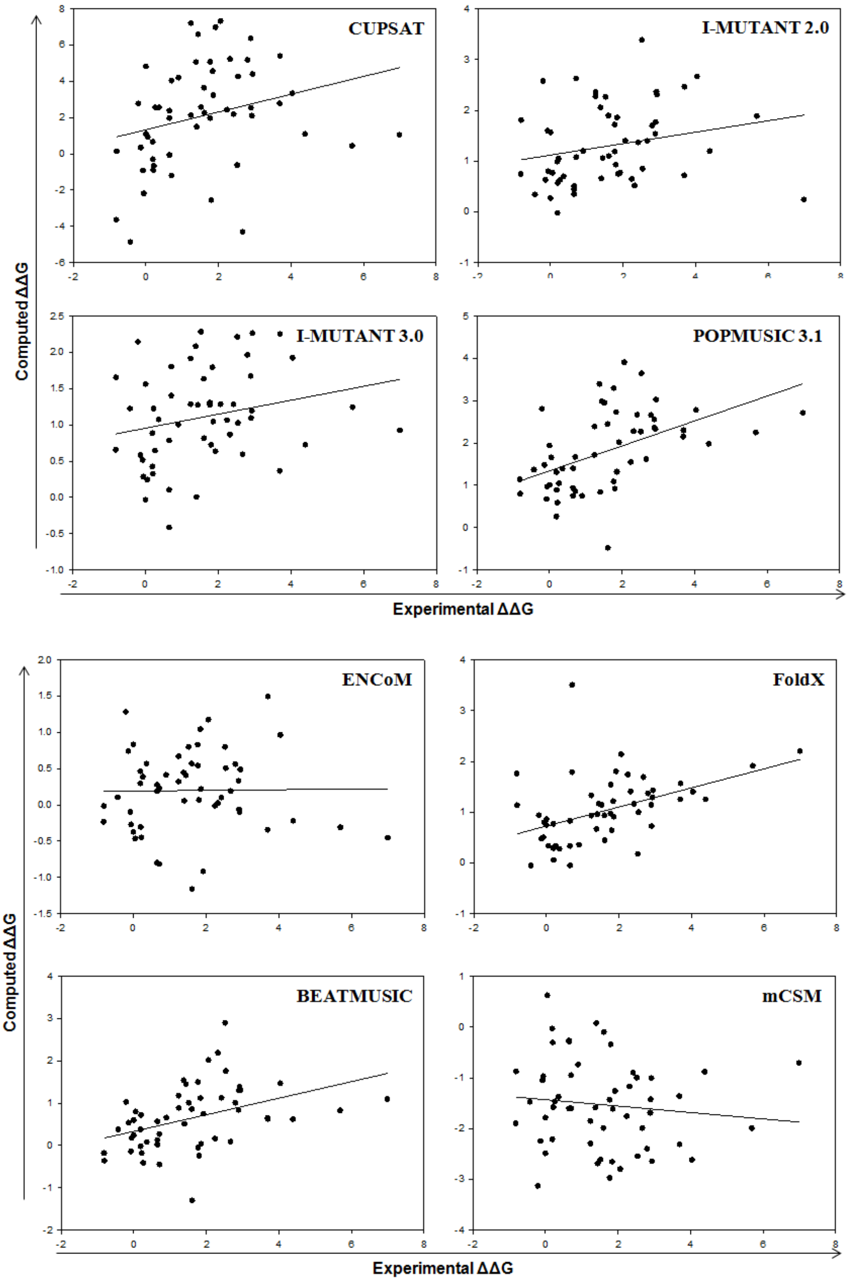

Figure S3. Correlation between experimental monomer and computed stability changes, ΔΔG (kcal/mol) for 54 mutants using 1HL4.

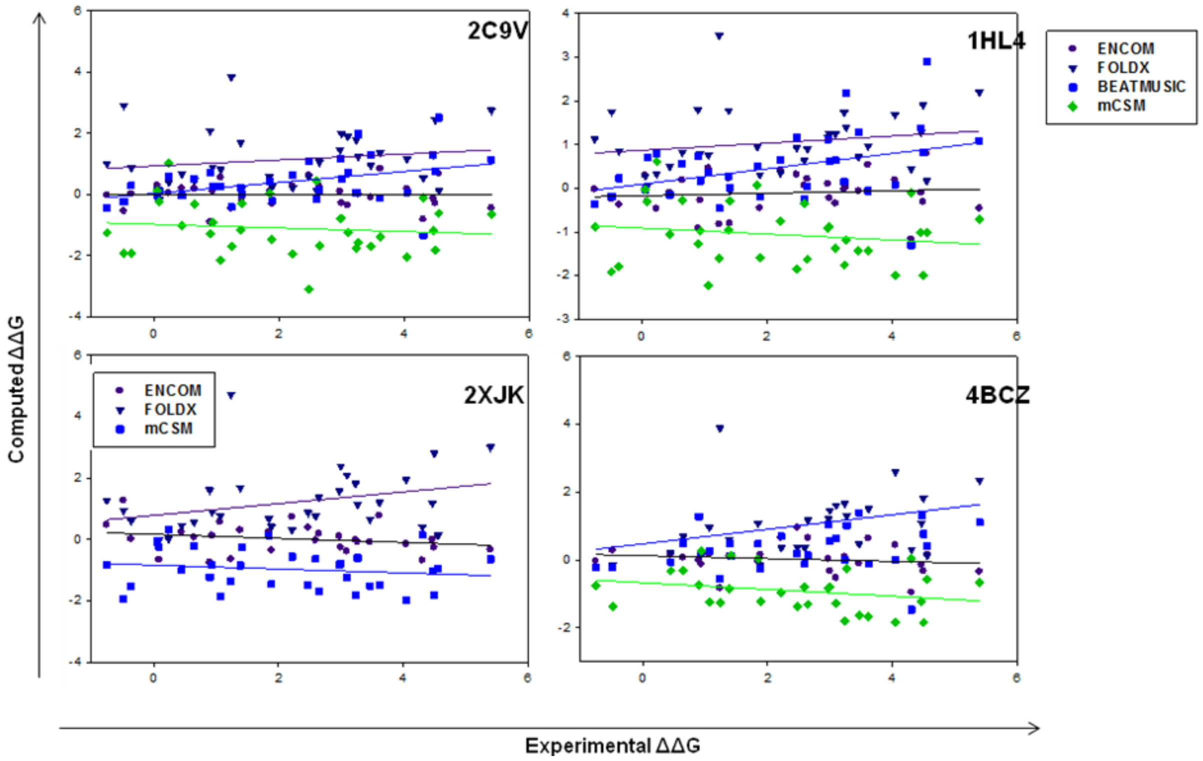

Figure S4. Correlation between experimental dimer and computed stability changes, ΔΔG (kcal/mol) for 33 mutants using four different structures.

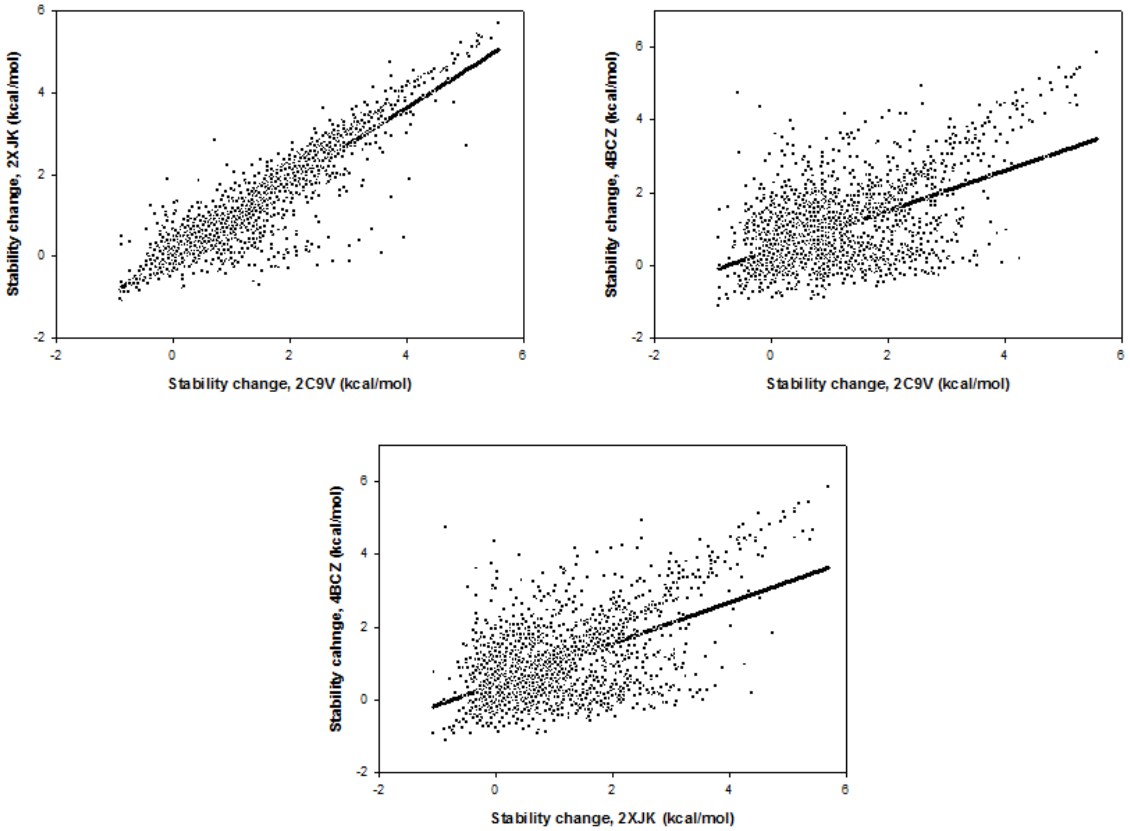

Figure S5: Correlation between predicted stability effects using PoPMuSiC 3.1 for all possible mutations in SOD1 using three different crystal structures (2C9V, 2XJK, and 4BCZ).

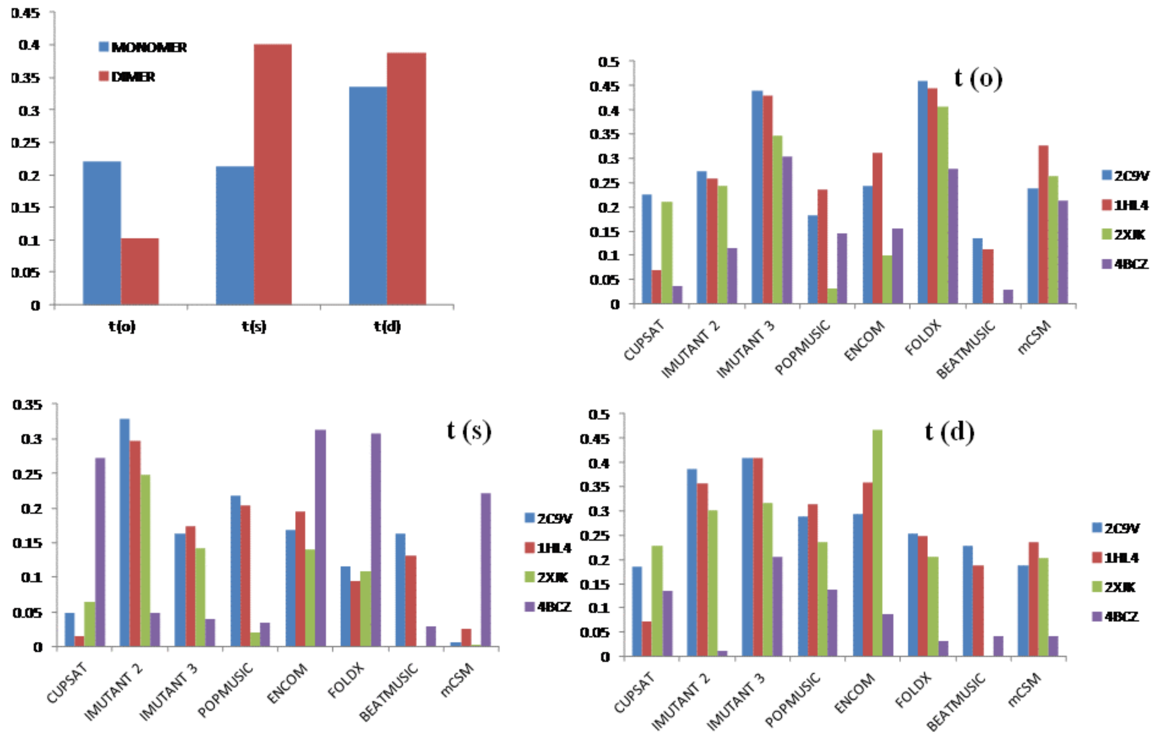

Figure S6. Comparative histograms for linear regression coefficient, R value between experimental ΔΔG values and normalized patients data for 30 mutations (See also Table 2).

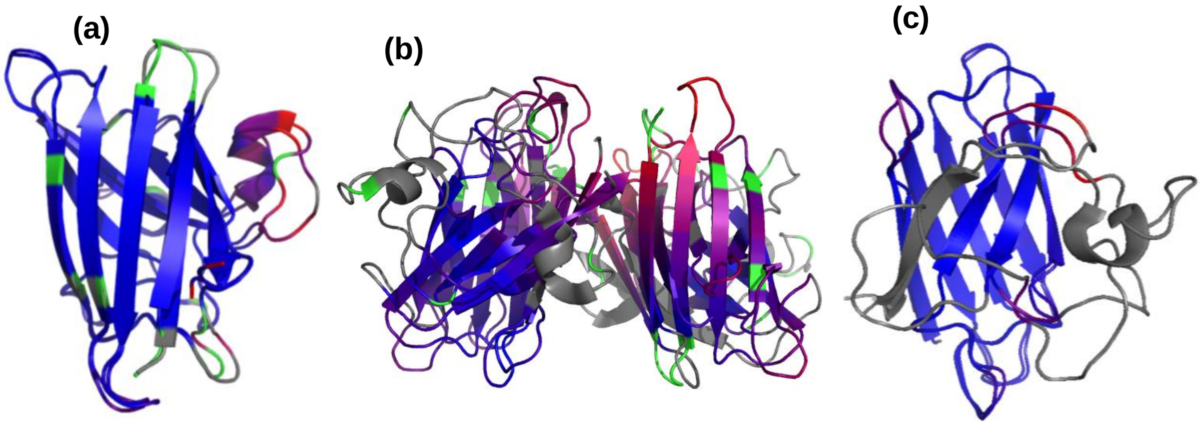

Figure S7. RMSD between different SOD1 structures. (a) 2C9V and 2XJK; (b) 2C9V and 4BCZ; (c) 2XJK and 4BCZ. Color coded are according to their RMSD values (Blue: low to Red: High).

References:

1 Lindberg, M. J., Bystrom, R., Boknas, N., Andersen, P. M. & Oliveberg, M. Systematically perturbed folding patterns of amyotrophic lateral sclerosis (ALS)-associated SOD1 mutants. *Proc Natl Acad Sci U S A* 102, 9754-9759, doi:0501957102 10.1073/pnas.0501957102 (2005).

2 Nordlund, A. & Oliveberg, M. Folding of Cu/Zn superoxide dismutase suggests structural hotspots for gain of neurotoxic function in ALS: parallels to precursors in amyloid disease. *Proc Natl Acad Sci U S A* 103, 10218-10223, doi:0601696103 10.1073/pnas.0601696103 (2006).

3 Bystrom, R., Andersen, P. M., Grobner, G. & Oliveberg, M. SOD1 mutations targeting surface hydrogen bonds promote amyotrophic lateral sclerosis without reducing apo-state stability. *J Biol Chem* 285, 19544-19552, doi:10.1074/jbc.M109.086074 (2010).

4 Stathopulos, P. B. *et al.* Calorimetric analysis of thermodynamic stability and aggregation for apo and holo amyotrophic lateral sclerosis-associated Gly-93 mutants of superoxide dismutase. *J Biol Chem* 281, 6184-6193, doi:M509496200 10.1074/jbc.M509496200 (2006).

5 Deng, H. X. *et al.* Amyotrophic lateral sclerosis and structural defects in Cu,Zn superoxide dismutase. *Science* 261, 1047-1051 (1993).

6 Aguirre, T., Matthijs, G., Robberecht, W., Tilkin, P. & Cassiman, J. J. Mutational analysis of the Cu/Zn superoxide dismutase gene in 23 familial and 69 sporadic cases of amyotrophic lateral sclerosis in Belgium. *Eur J Hum Genet* 7, 599-602, doi:10.1038/sj.ejhg.5200337 (1999).

7 Rainero, I. *et al.* SOD1 missense mutation in an Italian family with ALS. *Neurology* 44, 347-349 (1994).

8 Rosen, D. R. *et al.* Mutations in Cu/Zn superoxide dismutase gene are associated with familial amyotrophic lateral sclerosis. *Nature* 362, 59-62, doi:10.1038/362059a0 (1993).

9 DiDonato, M. *et al.* ALS mutants of human superoxide dismutase form fibrous aggregates via framework destabilization. *J Mol Biol* 332, 601-615, doi:S0022283603008891 (2003).

10 Andersen, P. M. *et al.* Phenotypic heterogeneity in motor neuron disease patients with CuZn-superoxide dismutase mutations in Scandinavia. *Brain* 120 (Pt 10), 1723-1737 (1997).

11 Segovia-Silvestre, T. *et al.* A novel exon 3 mutation (D76V) in the SOD1 gene associated with slowly progressive ALS. *Amyotroph Lateral Scler Other Motor Neuron Disord* 3, 69-74, doi:10.1080/146608202760196039 (2002).

12 Deng, H. X. *et al.* Two novel SOD1 mutations in patients with familial amyotrophic lateral sclerosis. *Hum Mol Genet* 4, 1113-1116 (1995).

13 Cao, X. *et al.* Structures of the G85R variant of SOD1 in familial amyotrophic lateral sclerosis. *J Biol Chem* 283, 16169-16177, doi:10.1074/jbc.M801522200 (2008).

14 Hayward, C., Brock, D. J., Minns, R. A. & Swingler, R. J. Homozygosity for Asn86Ser mutation in the CuZn-superoxide dismutase gene produces a severe clinical phenotype in a juvenile onset case of familial amyotrophic lateral sclerosis. *J Med Genet* 35, 174 (1998).

15 Beck, M., Sendtner, M. & Toyka, K. V. Novel SOD1 N86K mutation is associated with a severe phenotype in familial ALS. *Muscle Nerve* 36, 111-114, doi:10.1002/mus.20756 (2007).

16 Chou, C. M. *et al.* Identification of three mutations in the Cu,Zn-superoxide dismutase (Cu,Zn-SOD) gene with familial amyotrophic lateral sclerosis: transduction of human Cu,Zn-SOD into PC12 cells by HIV-1 TAT protein basic domain. *Ann N Y Acad Sci* 1042, 303-313, doi:1042/1/303 10.1196/annals.1338.053 (2005).

17 Andersen, P. M. *et al.* Autosomal recessive adult-onset amyotrophic lateral sclerosis associated with homozygosity for Asp90Ala CuZn-superoxide dismutase mutation. A clinical and genealogical study of 36 patients. *Brain* 119 (Pt 4), 1153-1172 (1996).

18 Hosler, B. A. *et al.* Three novel mutations and two variants in the gene for Cu/Zn superoxide dismutase in familial amyotrophic lateral sclerosis. *Neuromuscul Disord* 6, 361-366, doi:0960896696003537 (1996).

19 Banci, L. *et al.* SOD1 and amyotrophic lateral sclerosis: mutations and oligomerization. *PLoS One* 3, e1677, doi:10.1371/journal.pone.0001677 (2008).

20 Enayat, Z. E. *et al.* Two novel mutations in the gene for copper zinc superoxide dismutase in UK families with amyotrophic lateral sclerosis. *Hum Mol Genet* 4, 1239-1240 (1995).

21 Esteban, J. *et al.* Identification of two novel mutations and a new polymorphism in the gene for Cu/Zn superoxide dismutase in patients with amyotrophic lateral sclerosis. *Hum Mol Genet* 3, 997-998 (1994).

22 Kawata, A., Kato, S., Hayashi, H. & Hirai, S. Prominent sensory and autonomic disturbances in familial amyotrophic lateral sclerosis with a Gly93Ser mutation in the SOD1 gene. *J Neurol Sci* 153, 82-85, doi:S0022510X97001767 (1997).

23 Yulug, I. G., Katsanis, N., de Belleroche, J., Collinge, J. & Fisher, E. M. An improved protocol for the analysis of SOD1 gene mutations, and a new mutation in exon 4. *Hum Mol Genet* 4, 1101-1104 (1995).

24 Jones, C. T., Shaw, P. J., Chari, G. & Brock, D. J. Identification of a novel exon 4 SOD1 mutation in a sporadic amyotrophic lateral sclerosis patient. *Mol Cell Probes* 8, 329-330, doi:S0890-8508(84)71046-2 10.1006/mcpr.1994.1046 (1994).

25 Ikeda, M. *et al.* Variable clinical symptoms in familial amyotrophic lateral sclerosis with a novel point mutation in the Cu/Zn superoxide dismutase gene. *Neurology* 45, 2038-2042 (1995).

26 Zhang, H. *et al.* A rare Cu/Zn superoxide dismutase mutation causing familial amyotrophic lateral sclerosis with variable age of onset and incomplete penetrance in China. *Amyotroph Lateral Scler Other Motor Neuron Disord* 6, 234-238, doi:L24162GM3N14L426 10.1080/14660820510044478 (2005).

27 Gellera, C. *et al.* Superoxide dismutase gene mutations in Italian patients with familial and sporadic amyotrophic lateral sclerosis: identification of three novel missense mutations. *Neuromuscul Disord* 11, 404-410, doi:S0960896600002157 (2001).

28 Jacobsson, J., Jonsson, P. A., Andersen, P. M., Forsgren, L. & Marklund, S. L. Superoxide dismutase in CSF from amyotrophic lateral sclerosis patients with and without CuZn-superoxide dismutase mutations. *Brain* 124, 1461-1466 (2001).

29 Mase, G. *et al.* ALS with variable phenotypes in a six-generation family caused by leu144phe mutation in the SOD1 gene. *J Neurol Sci* 191, 11-18, doi:S0022510X01006256 (2001).

30 Sapp, P. C. *et al.* Identification of three novel mutations in the gene for Cu/Zn superoxide dismutase in patients with familial amyotrophic lateral sclerosis. *Neuromuscul Disord* 5, 353-357, doi:096089669500007A (1995).

31 Ghadge, G. D. *et al.* Mutant superoxide dismutase-1-linked familial amyotrophic lateral sclerosis: molecular mechanisms of neuronal death and protection. *J Neurosci* 17, 8756-8766 (1997).

32 Pramatarova, A. *et al.* Identification of new mutations in the Cu/Zn superoxide dismutase gene of patients with familial amyotrophic lateral sclerosis. *Am J Hum Genet* 56, 592-596 (1995).
